# Fisetin targets YB-1/RSK axis independent of its effect on ERK signaling: insights from *in vitro* and *in vivo* melanoma models

**DOI:** 10.1038/s41598-018-33879-w

**Published:** 2018-10-24

**Authors:** Mario Sechi, Rahul K. Lall, Saheed O Afolabi, Anant Singh, Dinesh C. Joshi, Shing-Yan Chiu, Hasan Mukhtar, Deeba N. Syed

**Affiliations:** 1 Department of Dermatology, University of Wisconsin, Madison, USA; 2 Department of Neuroscience, University of Wisconsin, Madison, USA; 3 Department of Chemistry and Pharmacy, University of Sassari, Sassari, Italy

## Abstract

The anti-proliferative activity of dietary flavonoid fisetin has been validated in various cancer models. Establishing its precise mechanism of action has proved somewhat challenging given the multiplicity of its targets. We demonstrated that YB-1 promotes epithelial-to-mesenchymal transition and its inhibition suppressed tumor cell proliferation and invasion. The p90 ribosomal S6 kinase (RSK), an important ERK effector, activates YB-1 to drive melanoma growth. We found that fisetin treatment of monolayer/3-D melanoma cultures resulted in YB-1 dephosphorylation and reduced transcript levels. In parallel, fisetin suppressed mesenchymal markers and matrix-metalloproteinases in melanoma cells. Data from cell-free/cell-based systems indicated that fisetin inhibited RSK activity through binding to the kinase. Affinity studies for RSK isoforms evaluated stronger interaction for RSK2 than RSK1. Competition assays performed to monitor binding responses revealed that YB-1 and RSK2 do not compete, rather binding of fisetin to RSK2 promotes its binding to YB-1. Fisetin suppressed YB-1/RSK signaling independent of its effect on ERK, and reduced MDR1 levels. Comparable efficacy of fisetin and vemurafenib for inhibiting melanoma growth was noted albeit through divergent modulation of ERK. Our studies provide insight into additional modes of regulation through which fisetin interferes with melanoma growth underscoring its potential therapeutic efficacy in disease progression.

## Introduction

Approximately 5 million patients are diagnosed with skin cancer in the United States, each year. Although melanoma is less common, it contributes to nearly 75% of skin cancer-related deaths^1^. A total of 67,753 people were diagnosed with invasive cutanoeus melanomas in the United States in 2012, the most recent year for which national data are available. More alarming are the statistics that show that, from the years 1975 to 2012, the incidence of melanoma has increased steadily at an annual average rate of 3.2% in men and 2.4% in women^1^. Thus, melanoma rates as the fifth and sixth most common cancer in men and women, respectively, and is reportedly one of the most common cancers among adolescents and young adults^1^. However, available treatment modalities applied so far have only a modest impact on overall survival once the disease has metastasized.

More than 90% of melanomas have increased activation of the mitogen-activated protein kinase (MAPK) pathway, with ~50% of patients displaying mutations in the BRAF and ~28% in NRAS kinases^2^. The p90 ribosomal S6 kinases (RSKs), downstream effectors of MAPK pathway, are serine/threonine protein kinases involved in the regulation of diverse cellular processes, such as growth, motility and survival. In humans, the RSK consists of four isoforms (RSK1, RSK2, RSK3 & RSK4), with 73 to 83% homology to each other. All share similar organization, comprising of two non-identical N-terminal (NTKD) and C-terminal (CTKD) kinase domains separated by a linker region of ~100 amino acids. The NTKD is responsible for substrate phosphorylation while the CTKD functions to regulate RSK activation via autophosphorylation^3^. It is thought that genes for two distinct protein kinases fused, generating a single kinase RSK, capable of receiving an upstream activating signal from ERK1/2 to its CTKD and transmitting an activating input to the NTKD^3^.

Several phosphorylation sites mapped within and outside of the RSK kinase domain, including serine^363^, serine^221^, serine^380^, threonine^359^ and threonine^573^ have been shown to be important for its activity^4^. The serine^363^ and serine^380^ residues are located in the linker region within the ‘turn motif’ and the ‘hydrophobic motif’ sequences of the kinase, respectively. The currently accepted model of RSK activation maintains that ERK1/2 activation results in the phosphorylation of threonine^573^ in the CTKD of RSK. The activated CTKD then autophosphorylates RSK at the serine^380^ residue. However, this site may also be phosphorylated by other kinases. In addition, ERK might also phosphorylate RSK at threonine^359^ and serine^363^ residues^5^. Alternatively, docking of PDKI at the phosphorylated hydrophobic motif phosphorylates serine^221^ in the NTKD activation loop resulting in RSK activation^4,5^.

RSK2 was found to be an essential regulator in tumor promoter induced cell transformation^6^. Activated RSK2 protein levels are highly abundant in human skin cancer tissues compared with normal skin^7^. Studies show that RSK through differential regulation of pro-apoptotic protein Bad mediates a MAPK-dependent tumor-specific survival signal in melanoma cells^8^. Others have demonstrated that stimulated ERK pathway reduces the sensitivity of melanoma cell lines to cisplatin through activation of RSK1^9^. Expression profiling analysis revealed that ERK-activated RSK induces transcription of an effective pro-motile invasive gene program which results in modulation of extracellular and the intracellular motility apparatus. Thus RSK serves as a key effector, from which multiple highly coordinate transcription-dependent mechanisms originate for stimulation of cell motility and invasion^10^. Given the fact that inhibition of RSK isoforms has proven effective in blocking invasion and metastasis, RSK inhibitors are being investigated for their specificity and selectivity in different tumor types^11^. More recently, RSK inhibition was shown to block cell proliferation and protein synthesis in BRAF/MEK inhibitor-resistant melanomas, establishing this pathway as a viable therapeutic strategy against chemoresistance^12^.

The dietary flavonoid fisetin is gaining much attention for its pleiotropic effects in various biological systems^13^. Fisetin has been studied for its neuroprotective activity and was shown to confer protection in multiple models of Huntington’s disease through ERK activation^14,15^. We and others have delineated its anti-cancer properties in several tumor models including melanoma and prostate^13^. We demonstrated that fisetin inhibits melanoma growth through suppression of Akt/mTOR pathways^16,17^. Analyzing the mechanism(s) of fisetin-induced cytotoxicity, we showed that fisetin mediated cell death is associated with induction of ER stress and activation of apoptotic pathways in human melanoma cells^18^. In other studies, fisetin was found to potentiate sorafenib-induced apoptosis and abrogate tumor growth in mice implanted with BRAF-mutant melanoma cells^19^. Fisetin suppressed human melanoma cell invasion by inhibiting epithelial-mesenchymal transition (EMT) and augmented anti-invasive and anti-metastatic potential of sorafenib in melanoma cells^20^. Recently, we identified YB-1, a DNA-RNA binding protein with a role in EMT and chemoresistance, as a key target of fisetin in prostate cancer cells^21^. Here we extended these studies and analyzed the mechanism through which fisetin exerts its effect on YB-1/RSK axis in human melanoma cells.

## Materials and Methods

### Materials

Fisetin was purchased from Sigma Chemical Co. (St. Louis, MO). FR180204 was obtained from Tocris Biosciences (Minneapolis, MN). BI-D1870 and vemurafenib were purchased from ApexBio (Houston, TX). Antibodies were obtained from Cell Signaling Technology (Danvers, MA).

### Cell culture/treatment

WM35 and A375 cells were obtained from ATCC (Manassas, VA), NCI/ADR-Res cells from NCI (Developmental Therapeutics Program, Frederick, MD) while 451Lu cells were provided by Dr. Meenhard Herlyn (Wistar Institute, PA). Authentication report was provided by ATCC and NCI for post-freeze viability, growth properties, morphology, mycoplasma contamination, species determination (cytochrome c oxidase I assay and STR analysis), sterility test and human pathogenic virus testing. Cells were frozen in aliquots in liquid nitrogen and cultured within six months. 451Lu cells were authenticated upon receipt using STR analysis and all cell lines are regularly tested for mycoplasma contamination using MycoAlert Mycoplasma Detection Kit from Lonza (Basel, Switzerland). WM35, A375 cells were cultured in DMEM, NCI/ADR-Res in RPMI and 451Lu in MEM with 10% FBS and 1% penicillin-streptomycin, at 37 °C with 5% CO_2_ in a humid environment. After treatment of cells with/without fisetin and/or inhibitors, whole cell lysates were prepared and western blot analysis was performed as described elsewhere^22^.

### Viability studies

Cells seeded in 24-well plates were treated at 70% confluence with/without fisetin and/or vemurafenib for 24 hours. Cell viability was determined by MTT assay as described^17^.

### Immunoprecipitation

Briefly, specific antibody was added to 100 μg of cell lysates after pre-clearing with protein A/G-agarose beads. After overnight incubation, protein A/G agarose beads were added and left for 3 h. After centrifugation and several washes, the pellet was resuspended with 2X sample buffer and electrophoresed on a SDS-PAGE gel.

### Immunochemistry

For immunohistochemistry, sections from melanoma constructs, fixed in 10% formalin were deparaffinized and rehydrated in xylene and a graded series of ethanol and blocked in 2% goat serum/PBS for 30 min. After incubation with specific primary antibody overnight and biotinylated secondary antibody for 1 h, slides were counterstained with hematoxylin and immunoreactive complexes were detected using 3,3′-diaminobenzidene (Dako Corp., CA). Immunocytochemical analysis was performed after seeding cells in 2-chamber tissue culture slides treated with/without fisetin and probing with fluorophore linked secondary antibodies^18^. Sections were visualized on Nikon Eclipse T*i* microscope and images captured with a camera attached to computer. Figures were composed using NIH ImageJ and Adobe photoshop 7.0 (Adobe Systems, CA).

### RNA isolation and qPCR

Total RNA was extracted from cells using RNeasy kit from Qiagen, (Germantown, MD), and reverse transcribed with iScript Reverse transcription supermix kit from BioRad (Hercules, CA). Fold change was calculated by the formula 2^−ΔΔ*CT*^ after normalization with GAPDH.

### Melanoma Skin Model

Full thickness melanoma skin model comprised of A375 melanoma cells, normal epidermal keratinocytes and fibroblasts was obtained from MatTek Corporation (Ashland, MA) and maintained at air-liquid interface. Fresh media was supplemented every alternate day with/without fisetin (80 μM) after which samples were fixed in 10% formalin for time-dependent studies^17^.

### Kinase binding assay

The KINOME*scan* screening platform (San Diego, CA) was employed to quantitatively measure interactions between fisetin and RSK isoforms. Binding constants (Kds) were calculated by measuring the amount of kinase captured on solid support as a function of the test compound concentration, using the Hill equation. The Hill Slope was set to −1. Curves were fitted using a non-linear least square fit with the Levenberg-Marquardt algorithm^17^.

### Kinase activity assay

*In vitro* kinase activity profiling of RSK isoforms was performed at Reaction Biology Corporation (Malvern, PA). Briefly, the specific kinase/substrate pair alongwith required cofactors was prepared in reaction buffer. Fisetin/staurosporine dissolved in DMSO was added to the mixture, followed by ^32^P-ATP. After incubation for 2 h, reactions were spotted onto filter paper which binds the radioisotope-labeled catalytic product^17^. After subtraction of background derived from control reactions containing inactive enzyme, kinase activity data was expressed as % remaining kinase activity.

### Surface plasmon resonance (SPR) binding assay

Binding experiments were performed using a Biacore T-200 instrument (Uppsala, Sweden) at 25 °C. 60 response units (RU) of the YB-1 human recombinant protein with GST-tag at N-terminal (51–139 aa, 35 kDa including GST tag, from Abnova Corporation, Taiwan) were directly immobilized on the EDC/NHS activated flow cell2 of the CM5 chip (GE certified) in water. The unoccupied sites were blocked with 1 M ethanol amine. The analytes RPS6KA1 (RSK1, 88.5 kDa) and RPS6KA3 (RSK2, 86.3 kDa) recombinant human proteins [full length and HIS-tag] (Life Technologies, Italy) were flown over the chip at variable concentrations in 10 mM sodium acetate buffer (pH 5.0), with flow rate of 5ul/min, at a single analyte concentration. Binding of analyte to the immobilized protein was monitored in real time to obtain on (ka) and off (kd) rates. The equilibrium constant (K_D_) was calculated from the observed ka and kd or by steady state kinetics as for the small molecules. Stocks were prepared in 100% DMSO, and further dilutions were made in assay buffer containing 10 mM HEPES buffer (pH 7.4), 150 mM NaCl, 3 mM EDTA, 0.05% P20 (polyoxyethylenesorbitan) and 0.1% BSA. Full kinetics analysis was performed at using analyte concentrations 50 nM (or 100 nM without BSA) to 0, run with serial dilutions, 50, 25, 12.5, 6.25, 3.125, 0 nM, and a flow rate of 30 ul/min. For competition assays, fisetin alone (1 µM and 10 µM) and RSK2 alone (100 nM) were flown over immobilized YB-1 to monitor binding response. The concentration of RSK2 was kept constant, but pre-incubated with increasing amounts of fisetin and then injected to determine the effect of fisetin on binding activity of RSK2 to YB-1.

### Efflux assay

As per manufacturer’s protocol (Millpore, Billerica, MA) A375 cells (2.5 × 10^5^ per group) were incubated with DiOC2(3) (15 min) or Rhodamine-123 (50 min) at 37 °C, after which fisetin (60 μM) or vinblastine (22 μM) was added. 30 min later cells were washed with cold efflux buffer and fluorescence was quantified on a Biotek plate reader at 488/530 nm.

### LC-MS/MS analysis

Vemurafenib concentration in fisetin-treated cells was determined by LC-MS/MS. An aliquot of cell lysate was spiked with PLX4720 (25 ng) [internal standard] and alkalinized by addition of two volumes of pH 11 buffer (1 mM sodium hydroxide and 0.5 mM sodium bicarbonate). Organic layer was separated and dried under nitrogen. Samples were reconstituted in 100 μl of mobile phase and transferred into HPLC glass vials. Chromatographic analysis was performed using the AQUITY UPLC system (Waters, Milford, MA). Chromatographic separation was achieved using an Agilent Technologies Eclipse XDB-C18 column (4.6 × 50 mm) with 1.8-μm Zobrax Rx-SIL as the stationary phase. Mobile phase consisted of 20 mM ammonium formate with 0.1% formic acid and acetonitrile (30:70 v/v), delivered at a flow rate of 0.25 ml/min. Samples were analyzed using an electrospray probe in the negative ionization mode operating at a voltage of 2.96 kV for both vemurafenib and PLX4720. The assay was sensitive and linear over a range of 1.2 ng/ml-1.2 μg/ml, with the coefficient of variation being less than 15% over the entire range.

### Xenograft studies

Athymic (nu/nu) female nude mice (Harlan, Madison, WI), 4–5 weeks old, were housed under pathogen-free conditions, in the University of Wisconsin Animal Resource Facility, with a 12-hour light/12-hour dark schedule and fed an autoclaved diet *ad libitum*. All animal experiments were approved by and carried out according to Institutional Animal Care and Use Committee guidelines, University of Wisconsin-Madison, protocol # M005212. Briefly, 1 × 10^6^ 451Lu cells were injected s/c into each flank of 18 mice randomly divided into 3 groups. The first group received intraperitoneal injection of fisetin 40 mg/kg b.wt. (1 mg/animal) thrice weekly, the second group received vemurafenib by oral gavage 80 mg/kg b.wt. (2 mg/animal) dissolved in DMSO, 5 days/week, while the third group served as the DMSO treated control group. Mice weight and tumor volumes were recorded twice weekly. The animals were sacrificed when tumors reached a volume of 1200 mm^3^ in the control group. Samples were collected and stored at −80 °C until further analysis.

### Statistical analysis

Results were analyzed using a two-tailed Student’s unpaired *t* test, using GraphPad QuickCals software (unless indicated otherwise) and *p < 0.05, **p < 0.01 and ***p < 0.001 were considered statistically significant.

## Results

### Fisetin inhibits YB-1 in BRAF mutant melanoma cells

**Figure 1:**
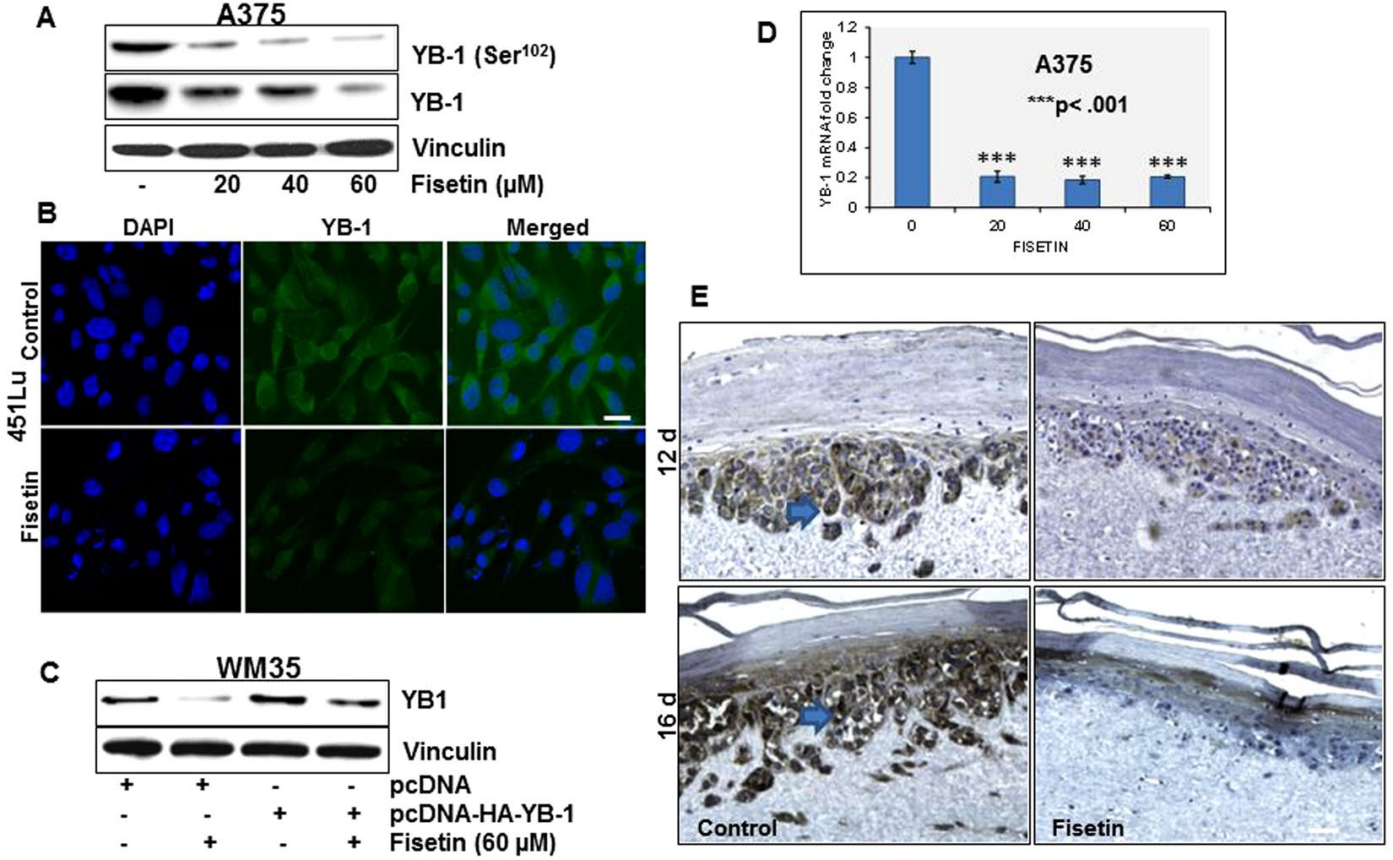
Fisetin inhibits YB-1 in BRAF mutant melanoma cells (**A**) Whole cell lysates of A375 melanoma cells treated with fisetin (20–60 µM:24 h) were analyzed for p-YB-1 and YB-1 protein expression. Equal loading was confirmed by reprobing for vinculin. (**B**) Representative micrographs (20x) showing immunofluorescence for YB-1 in 451Lu melanoma cells with/without fisetin (60 µM:24 h). Scale bar, 20 µm. (**C**) WM35 melanoma cells transfected with pcDNA-HA-YB-1 and treated with fisetin (60 µM:24 h) were analyzed for YB-1 expression. (**D**) Histogram represents relative YB-1 mRNA levels in fisetin-treated A375 cells (20–60 µM:24 h) normalized to GAPDH. (**E**) Representative micrographs (10x) showing YB-1 expression in A375 melanoma constructs treated with fisetin (80 µM), harvested at day 12 and 16 post treatment. Scale bar, 20 µm.

YB-1 expression is reportedly upregulated during melanoma progression and has been linked to proliferation, invasion and resistance to apoptosis^23^. We examined the effect of fisetin on endogenous levels of YB-1 in highly aggressive BRAF mutant melanoma cells. Fisetin treatment (0–80 µM:24 h) to A375 and 451Lu melanoma cells resulted in a dose-dependent decrease in YB-1 phosphorylation concomitant with downregulation of total protein levels, as assessed by western blot and immunocytochemical analysis (Fig. 1A,B, Sup. Fig. 1A–C). We selected WM35 melanoma cells, representative of non-invasive radial growth phenotype with an intrinsically low YB-1 expression for overexpression studies (Sup. Fig. 1D). A similar effect was observed in WM35 melanoma cells where exogenous YB-1 levels were significantly decreased with fisetin treatment (Fig. 1E). We then examined the effect of fisetin on YB-1 mRNA levels. qPCR analysis showed a significant reduction in YB-1 transcript levels in fisetin-treated A375 cells (Fig. 1D). Finally, we studied the expression of YB-1 in 3-D melanoma constructs at different stages of melanoma progression. Immunohistochemical analysis of untreated melanoma constructs showed increased expression of YB-1 which correlated with melanoma progression. In contrast, fisetin-treated melanoma constructs, at both time points (days 12 and 16) showed diminished staining of YB-1 versus the untreated controls (Fig. 1E). We had previously shown that forced expression of YB-1 promoted EMT in prostate cancer that was effectively inhibited by fisetin^21^. We examined EMT markers in fisetin-treated A375 melanoma cells and observed a decrease in vimentin and N-cadherin accompanied by an increase in E-cadherin. Moreover, fisetin treatment resulted in downregulation of MMP-2 and MMP-9 (data not shown) in monolayer and 3-D melanoma cultures (Sup. Fig. 1F,G).

### Fisetin binds to RSK and suppresses its kinase activity

**Figure 2:**
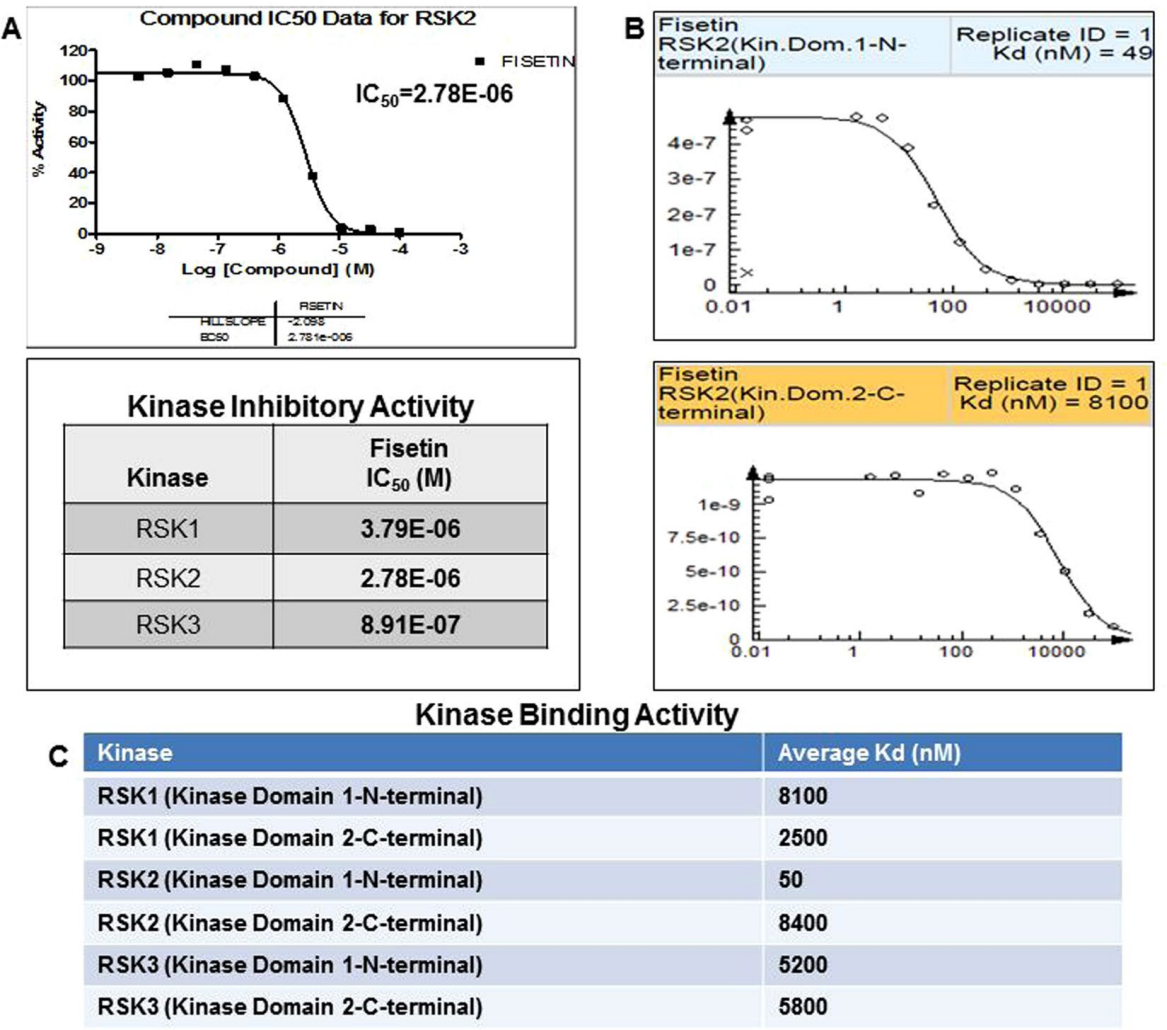
Fisetin binds to RSK and suppresses its kinase activity (**A**) Kinase inhibitory activity: (top) Representative curve of % kinase inhibitory activity for RSK2. (bottom) % kinase inhibitory activity tabulated for RSK isoforms. Fisetin was tested for kinase activity inhibition against RSK1, RSK2 and RSK3 in 10-dose IC_50_ mode with 3-fold serial dilution starting at 100 μM. Reactions were carried out at 30 μM ATP. (**B**) Kinase binding assay: Representative curves of competition binding assay for RSK2, N and C termini. An 11-point 3-fold serial dilution of fisetin was prepared in 100% DMSO at 100X final test concentration and diluted to 1X in the assay. Kds were determined using a top concentration of 100 µM. The amount of kinase measured by qPCR (signal; *y*-axis) is plotted against the corresponding compound concentration in log10 scale (*x*-axis). (**C**) Binding constants for N and C termini of each RSK isoform obtained from kinase binding studies are tabulated where each experiment was performed in duplicate.

RSK has been recognized as a novel activator of YB-1, able to phosphorylate its serine^102^ residue^24^. To examine if suppression of YB-1 phosphorylation by fisetin is linked to its inhibitory effect on RSK, we utilized radiometric assays (Reaction Biology Corp) to measure the kinase catalytic activity of RSK isoforms. Comparative studies in the presence of ^32^P-γ-ATP showed that fisetin inhibited RSK3>RSK2>RSK1 with IC_50_ values ranging from 8.91E-07, 2.78E-06 and 3.79E-06 M, respectively (Fig. 2A, Sup. Fig. 2A). Employing the Kinome*scan* screening platform, ATP site-dependent competition binding assays were performed to quantitatively measure interactions between fisetin and RSK. Screening hits were identified by measuring the amount of kinase captured in treated versus control samples using qPCR, subsequent to which dissociation constants (Kds) for compound-kinase interactions were calculated. Remarkably, fisetin exhibited the highest affinity for RSK2 at its NTKD (50 nM) while the Kds for RSK1 and RSK3 ranged from 8100:2500 and 5200:5800 nM for N and C-termini, respectively (Fig. 2B,C, Sup. Fig. 2B).

### Fisetin/RSK2 complex augments its binding to YB-1

**Figure 3:**
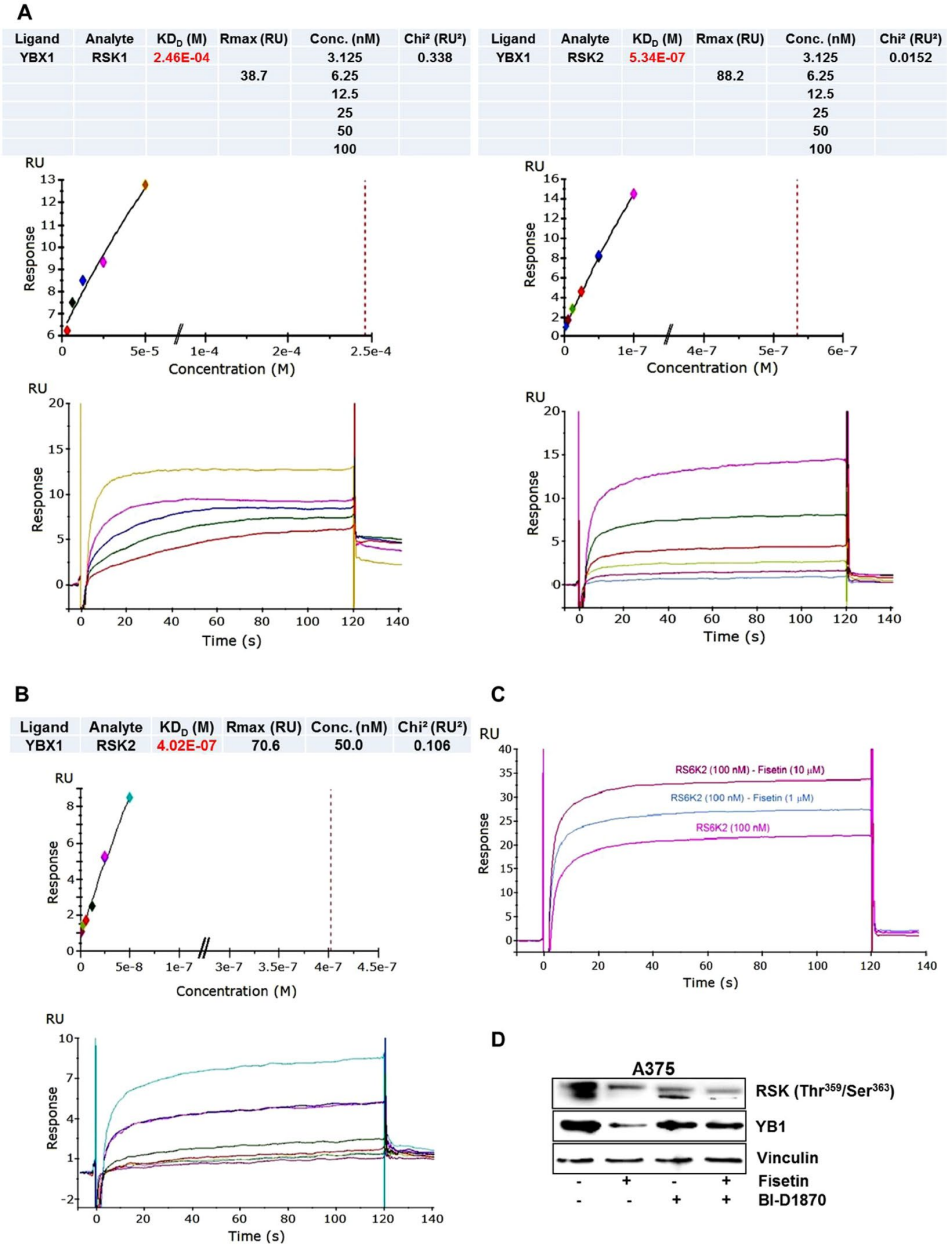
Fisetin/RSK2 complex augments its binding to YB-1 (**A**) Representative SPR sensorgrams showing full kinetics for direct binding characteristics between YB-1 and RSK1 (left) or YB-1 and RSK2 (right). Briefly, YB-1 was directly immobilized on the sensor chip by amine coupling and RSK1 or RSK2 was flown over the protein-coated chip at different concentrations (100-0 nM). ka and kd or steady state kinetics were used for determining the K_D_. Chi square (χ^2^) analysis was carried out between the actual sensorgram (colored line, bottom) and the sensorgram generated from the BIAnalysis software (black line, top) to determine the accuracy of the analysis. χ^2^ value within 1–2 was considered significant (accurate), and below 1 was highly significant (highly accurate). (**B**) Representative sensorgram showing full kinetics between YB-1 and RSK2. In the presence of BSA, YB-1 was directly immobilized on the sensor chip by amine coupling and RSK2 was flown over the protein-coated chip at different concentrations (50-0 nM). (**C**) Representative sensorgram for competition assays, where fisetin and RSK2 were flown over immobilized YB-1. The concentration of RSK2 was kept constant (100 nM), but pre-incubated with increasing amounts of fisetin (1 and 10 µM) and then injected to determine the effect of fisetin on binding activity of the RSK2 to YB-1. Data for affinity evaluation were obtained from a concentration dependent binding curve for the interaction of increasing amounts of fisetin with constant concentration of RSK2. (**D**) Whole cell lysates of fisetin-treated A375 melanoma cells (60 μM:24 h) with/without RSK inhibitor BI-D1870 were analyzed for YB-1 expression. Equal loading was confirmed by reprobing for vinculin.

Our *in silico* studies indicated that fisetin binds to YB-1 on residues β1-β4 strands within its cold shock domain^21^. To examine if binding of fisetin to RSK isoforms had any impact on its binding to YB-1, we performed real-time interaction analysis using SPR assays. Binding evaluation was conducted by immobilizing the YB-1 protein on a sensor surface. The analytes (RSK1/RSK2) were then injected in solution over the surface. Changes in SPR response were analyzed, providing kinetic and affinity data. The scouting full kinetics performed to evaluate binding between YB-1 and RSK1/YB-1 and RSK2 indicated stronger interactions for the latter (K_D_ = 2.46E-04M for RSK1 vs 5.34E-07M for RSK2, respectively) (Fig. 3A). Subsequent full kinetic assays repeated for RSK2/YB-1 in the presence of BSA, yielded K_D_ values very close to those without BSA (4.02 × −07M) validating the system (Fig. 3B). Competition assays were carried out flowing first fisetin alone and then RSK2 alone over immobilized YB-1 to monitor the binding response (data not shown). In the next step, RSK2 and fisetin were preincubated, and binding experiments were finalized (Fig. 3C). Contrary to our expectations, data obtained revealed that RSK2 and YB-1 do not compete with each other; rather binding of fisetin to RSK2 seems to promote its binding to YB-1. Reverting to a cell-based system, we treated A375 melanoma cells with RSK inhibitor BI-D1870 with or without fisetin and scrutinized YB-1 expression. Both fisetin and BI-D1870 had similar effect on the phosphorylation of RSK at 359/363 residues. However, in comparison to fisetin BI-D1870 had only a modest effect on YB-1 expression (Fig. 3D, Sup. Fig. 3). This is in agreement with previous observations where BI-D1870 decreased phosphorylation of YB-1 but total protein levels remained unaltered^25^. In our studies, the combinatorial treatment did not yield an additive decrease in YB-1 levels. Taken together, the data suggest that formation of YB-1/RSK complex may be an important regulatory event in fisetin-mediated decrease of YB-1.

### Fisetin induced modulation of YB-1/RSK signaling is associated with decrease in MDR1

**Figure 4:**
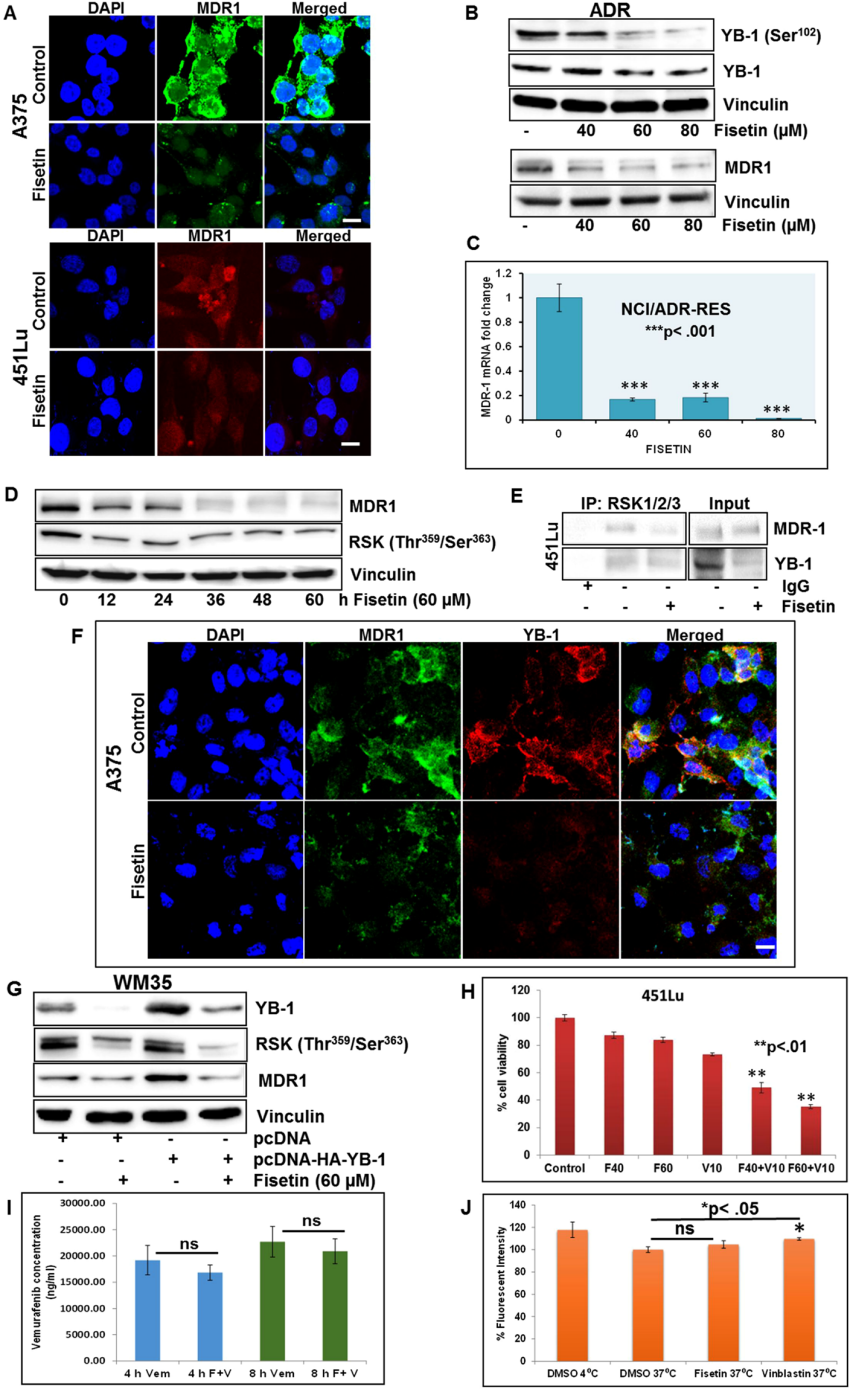
Fisetin induced modulation of YB-1/RSK signaling is associated with decrease in MDR1 (**A**) Representative images (20x) showing immunofluorescence for MDR1 in fisetin-treated A375 and 451Lu melanoma cells. Scale bar, 20 µm. DAPI was used as nuclear staining control. (**B**) Whole cell lysates of NCI/ADR-Res ovarian cancer cells treated with fisetin (40–80 µM:24 h) were analyzed for p-YB-1, YB-1 and MDR1 expression. Equal loading was confirmed by reprobing for vinculin. (**C**) Histogram represents relative MDR1 levels in NCI/ADR-Res cells treated with fisetin (40–80 µM:24 h). Gene expression was measured by qPCR and normalized to GAPDH. Error bars represent mean ± SE among three independent experiments, where each experiment was performed in triplicate. (**D**) Analysis of MDR1 and p-RSK in fisetin-treated A375 cells at specified time points. Equal loading was confirmed by reprobing for vinculin. (**E**) Equal amounts of cell lysates treated with/without fisetin (60 μM:24 h) were immunoprecipitated with RSK antibody followed by western blot analysis for MDR1 and YB-1 antibodies. (**F**) Representative images (20x) showing immunofluorescence for MDR1 (green) and YB-1 (red) in fisetin-treated (60 µM:24 h) A375 melanoma cells. Scale bar, 20 µm. (**G**) WM35 melanoma cells transfected with pcDNA-HA-YB-1, treated with fisetin (60 µM:24 h) were analyzed for p-RSK and MDR1 expression. Equal loading was confirmed by reprobing for vinculin. (**H**) Viability studies in 451Lu melanoma cells, 24 post treatment with/without fisetin and vemurafenib, as assessed by MTT assay. (**I**) Intracellular vemurafenib concentration in A375 melanoma cells with/without fisetin treatment (60 μM) at 4 and 8 hours, quantified by LC-MS/MS analysis. (**J**) A375 cells loaded with DiOC2(3) and incubated at 37 °C with/without vinblastine or fisetin were quantified on Biotek plate reader, where DMSO at 4 °C served as positive control. Data shown are representative of three independent experiments with similar results.

RSK protects MDR1 against ubiquitin-mediated proteasomal degradation^26^. Moreover, increase in MDR1 expression has been linked to YB-1 phosphorylation and nuclear translocation in various tumor models^27^. Our studies demonstrated significantly diminished MDR1 levels in A375 and 451Lu melanoma cells (Fig. 4A, Sup. Fig. 4A). The NCI/ADR-Res is an ovarian tumor cell line that expresses high levels of MDR1. We therefore examined the susceptibility of NCI/ADR-Res cells to fisetin. Consistent with our previous data, these cells showed reduced viability and decrease in MDR1 and YB-1 levels upon treatment with fisetin (Fig. 4B,C, Sup. Fig. 4B–D). Time course analysis in A375 cells revealed decreased MDR1 protein expression as early as 12 h post treatment. A parallel decrease in RSK phosphorylation at threonine^359^/serine^363^ residues was observed (Fig. 4D, Sup. Fig. 4E). Evaluation of fisetin-treated 451Lu and NCI/ADR-Res cell lysates, incubated with agarose beads coated with antibody against RSK validated the presence of YB-1/RSK complex and interaction between RSK and MDR1 (Fig. 4E, Sup. Fig. 4F). Immunocytochemical analysis demonstrated enhanced staining of YB-1 and MDR1 in untreated melanoma cells that was significantly reduced with fisetin treatment (Fig. 4F). To further study these interactions, we overexpressed YB-1 in WM35 melanoma cells and assessed MDR1 expression. Interestingly, YB-1 overexpression increased MDR1 expression yet had little effect on phosphorylated RSK levels suggestive of a hierarchy where YB-1 functions downstream of RSK. Regardless, fisetin treatment abrogated RSK phosphorylation at threonine^359^/serine^363^ and decreased MDR1 levels in YB-1 overexpressing WM35 melanoma cells (Fig. 4G, Sup. Fig. 4G).

We hypothesized that the strong suppressive effect of fisetin on MDR1 expression should potentially translate into potent inhibition of MDR1 substrate efflux, enhanced intracellular accumulation and therapeutic efficacy. To corroborate this line of thought, we tested whether fisetin can augment the inhibitory effect of BRAF inhibitor vemurafenib (PLX4032), a known MDR1 substrate. After establishing the IC_50_ of vemurafenib (Sup. Fig. 4H) and fisetin (data not shown) in melanoma cell lines, we evaluated the combinatorial effect of vemurafenib and fisetin on melanoma cell viability. Our studies showed that combination of fisetin and vemurafenib was significantly more potent in decreasing cell viability in both A375 (data not shown) and 451Lu melanoma cells when compared to treatment with each compound alone (Fig. 4H). We then asked if the increased efficacy of the combination was due to increased cellular accumulation of vemurafenib, due to the inhibitory effect of fisetin on MDR1 mediated drug efflux. To answer this question, 451Lu (data not shown) and A375 melanoma cells, harvested 4 h and 8 h post treatment with fisetin and vemurafenib, were analyzed by LC/MS/MS (Fig. 4I). The combination group compared to cells treated with vemurafenib alone did not have any appreciable increase in vemurafenib concentration. To further study the functional relevance of fisetin-mediated MDR1 inhibition, we evaluated the efflux of MDR1 substrates DiOC2(3) and rhodamine in A375 melanoma and NCI/ADR-Res ovarian cancer cells under physiologic conditions. The relative fluorescence of cell populations treated with fisetin or known MDR1 inhibitors was quantified. Increased fluorescence in cells treated with MDR1 inhibitor vinblastine, indicated effective blockage of DiOC2(3) and rhodamine (data not shown) efflux. Fisetin on the other hand had only a modest effect on blocking the efflux, and the increase in fluorescence was not significant when compared to DMSO treated cells (Fig. 4J). This indicated that fisetin is not effective in inhibiting the efflux of MDR1 substrates, despite its suppressive effect on RSK/YB-1/MDR1 signaling axis.

### Fisetin mediated decrease in RSK/YB-1 does not require suppression of ERK signaling

**Figure 5:**
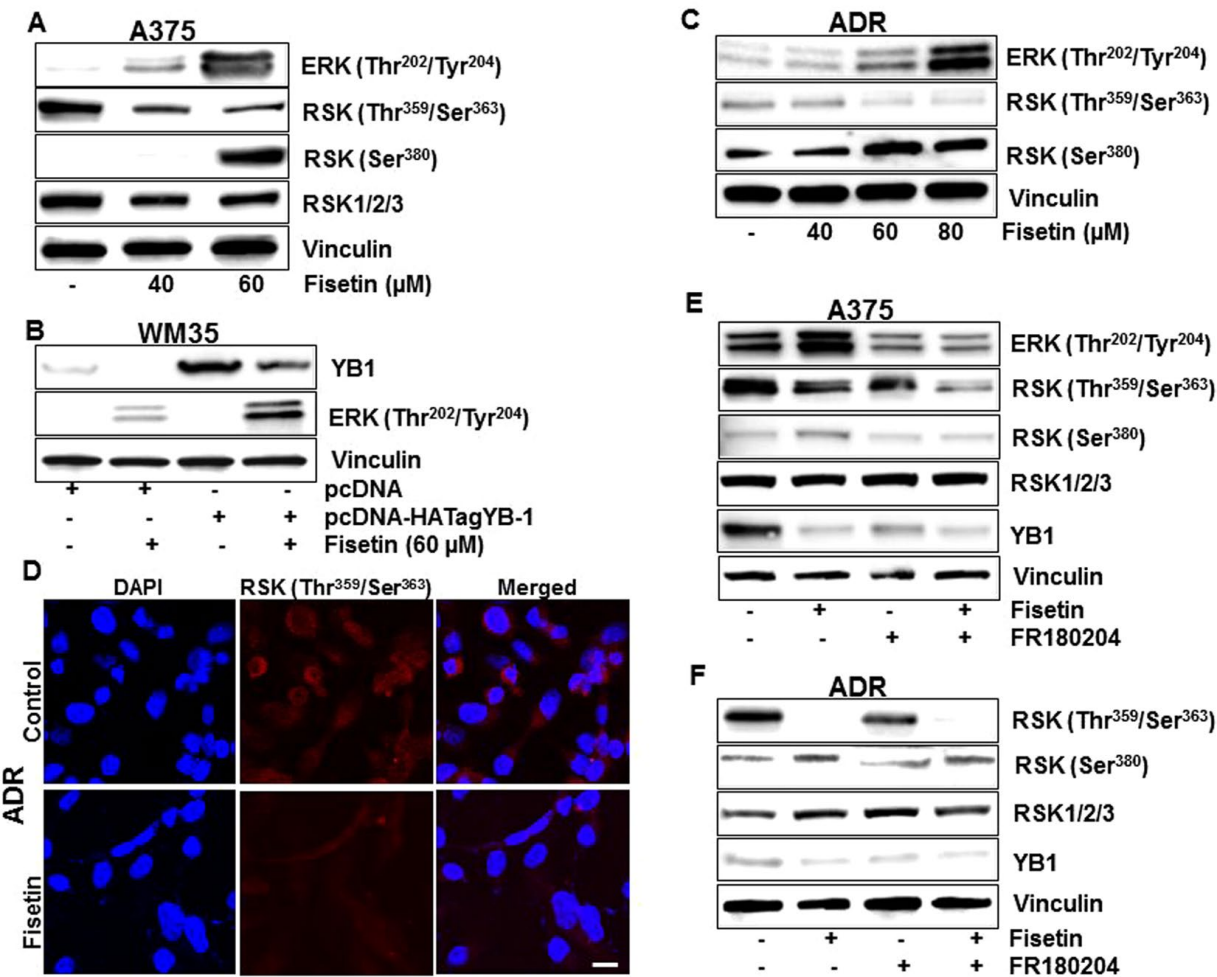
Fisetin mediated decrease in RSK/YB-1 does not require suppression of ERK signaling (**A**) Whole cell lysates of A375 cells treated with fisetin (40–60 µM:24 h) were analyzed for ERK1/2 and RSK protein expressions. Equal loading was confirmed by reprobing for vinculin. (**B**) WM35 melanoma cells, transfected with pcDNA-HA-YB-1, treated with fisetin (60 µM:24 h) were analyzed for ERK1/2 expression. Equal loading was confirmed by reprobing for vinculin. (**C**) Whole cell lysates of NCI/ADR-Res ovarian cancer cells treated with fisetin (40–80 µM:24 h) were analyzed for phosphorylated RSK and ERK1/2 protein expressions. Equal loading was confirmed by reprobing for vinculin. (**D**) Representative images (20x) showing immunofluorescence for p-RSK (red) in fisetin-treated (60 µM:24 h) NCI/ADR-Res cells. Scale bar, 20 µm. (**E,F**) Whole cell lysates of A375 melanoma and NCI/ADR-Res ovarian cancer cells, treated with fisetin (60 µM:24 h) and/or ERK1/2 inhibitor FR180204 (10 µM) were analyzed for phosphorylated RSK and YB-1 protein expressions. Equal loading was confirmed by reprobing for vinculin. Data shown are representative of three independent experiments with similar results.

Given the regulatory role of ERK signaling on RSK/YB-1 axis and its importance in BRAF mutated melanoma, we examined the effect of fisetin on ERK phosphorylation. Intriguingly, 24 h post treatment, fisetin increased the phosphorylation of ERK1/2 at threonine^202^/tyrosine^204^ residues in 451Lu (data not shown) and A375 melanoma cells, at doses linked to decreased cell viability (Fig. 5A, Sup. Fig. 5A). Moreover, fisetin treated YB-1-overexpressing WM35 melanoma cells (Fig. 5B, Sup. Fig. 5B) and chemoresistant NCI/ADR-Res ovarian cancer cells (Fig. 5C, Sup. Fig. 5C) also exhibited a dose dependent increase in ERK phosphorylation at 24 h. We further observed that fisetin mediated dose-dependent decrease in RSK phosphorylation at threonine^359^/serine^363^ residues (Fig. 5A,C,D) was accompanied with increased phosphorylation at the serine^380^ residue (Fig. 5A,C, Sup. Fig. 5A–C). To investigate if RSK phosphorylation was regulated by ERK, A375 melanoma cells were exposed to FR180204, a selective ERK1/2 inhibitor. A significant decrease in RSK phosphorylation at threonine^359^/serine^363^ residues was observed when cells were treated with fisetin or FR180204. Inhibition of ERK did not interfere with RSK phosphorylation at threonine^359^/serine^363^ residues in fisetin-treated cells (Fig. 5E,F, Sup. Fig. 5D,E). However, fisetin-mediated increase in serine^380^ phosphorylation was abrogated with ERK inhibition suggesting that ERK was driving phosphorylation at this residue (Fig. 5E, Sup. Fig. 5D,E). Importantly, fisetin mediated decrease in YB-1 levels was unaffected by ERK inhibition (Fig. 5E,F). Thus, dephosporylation of RSK at threonine^359^/serine^363^ residues seems to be an important determinant in fisetin mediated inhibition of YB1, independent of ERK signaling.

### Comparable inhibition of melanoma tumor growth by fisetin and vemurafenib albeit through divergent regulation of ERK signaling

**Figure 6:**
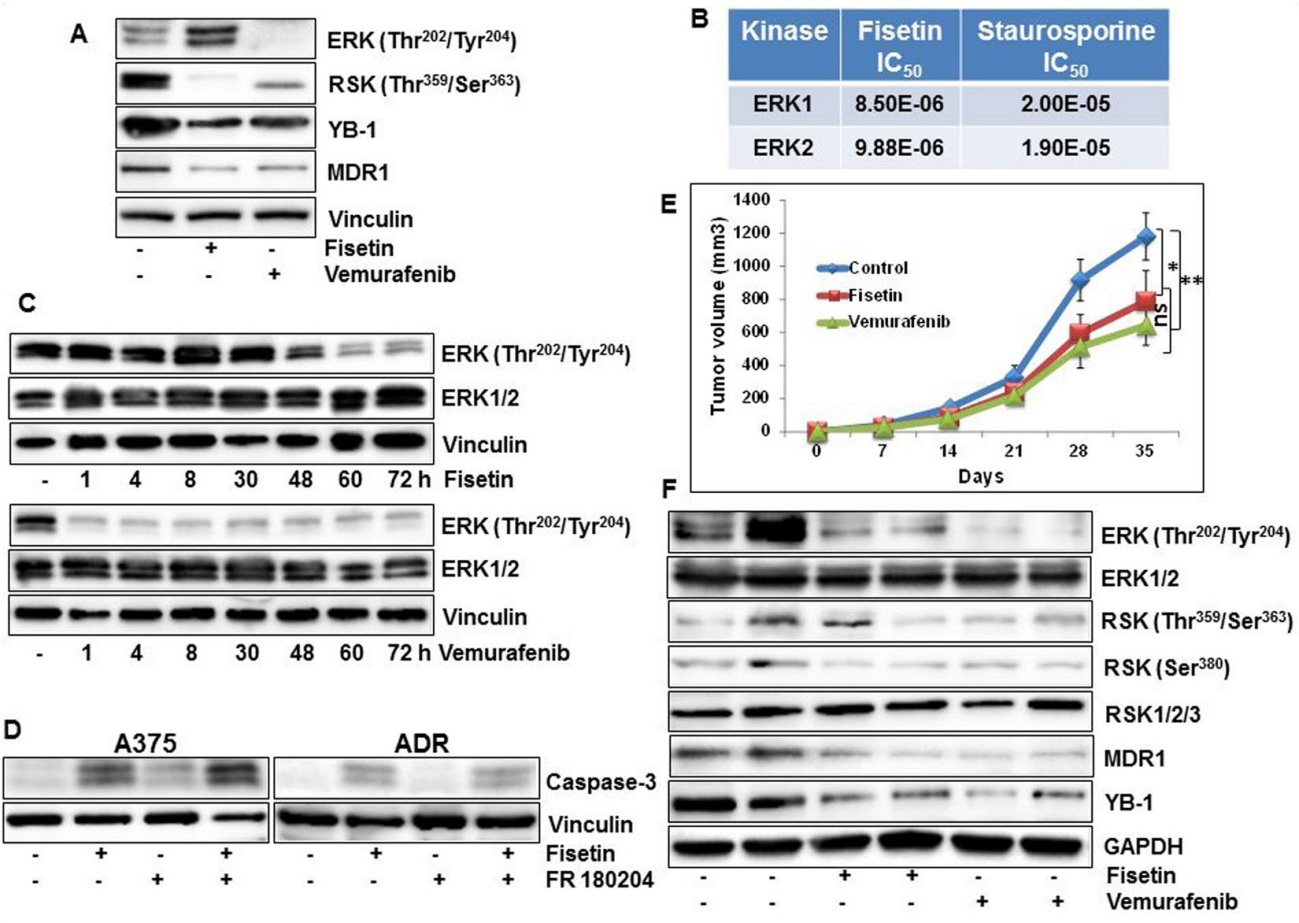
Comparable inhibition of melanoma tumor growth by fisetin and vemurafenib albeit through divergent regulation of ERK signaling (**A**) Whole cell lysates of A375 melanoma cells treated with fisetin (60 µM) or vemurafenib (10 µM) for 24 h were analyzed for phosphorylated RSK, ERK1/2, total YB-1 and MDR1. Equal loading was confirmed by reprobing for vinculin. (**B**) Kinase inhibitory activity for ERK1/2. Fisetin was tested in 10-dose IC_50_ mode with 3-fold serial dilution starting at 100 μM. Reactions were carried out at 30 μM ATP. (**C**) Whole cell lysates of A375 melanoma cells treated with fisetin (60 µM) or vemurafenib (10 µM) for specified time points analyzed for phosphorylated and total ERK1/2. Equal loading was confirmed by reprobing for vinculin. (**D**) Whole cell lysates of A375 melanoma and NCI/ADR-Res ovarian cancer cells, treated with fisetin (60 µM) and/or ERK1/2 inhibitor FR180204 (10 µM) for 24 h were analyzed for cleaved caspase-3 expression. Equal loading was confirmed by reprobing for vinculin. (**E**) Average tumor volume of DMSO/fisetin/vemurafenib-treated mice plotted over days after tumor cell inoculation. Points, mean of 12 tumors in six animals; bars, mean ± SE,*p ≤ 0.05, **p ≤ 0.01 versus the control group. (**F**) Whole cell lysates of tumor tissues analyzed by western blot. Equal loading was confirmed by reprobing for GAPDH. Data represent samples from each group repeated twice with similar results.

The biochemical affinity of vemurafenib for mutated BRAF translates into potent suppression of ERK phosphorylation and inhibition of cell proliferation^28^. We compared the effect of fisetin and vemurafenib on RSK/YB1 signaling in *in vitro* melanoma cultures. A375 cells treated with vemurafenib for 24 h showed a comparable decrease in YB-1 expression and RSK phosphorylation levels as witnessed with fisetin treatment. In contrast to vemurafenib, fisetin treatment induced ERK1/2 phosphorylation in A375 melanoma cells (Fig. 6A, Sup. Fig. 6A). However, cell free kinase activity studies indicated that fisetin mediated inhibition of ERK1/2 kinase activity was greater than staurosporine, used as a positive control (Fig. 6B, Sup. Fig. 6B). To evaluate this apparent discrepancy we performed a time course analysis of fisetin-treated A375 cells. We observed that an initial increase in ERK1/2 phosphorylation was followed by a sustained decrease, suggestive of a biphasic response (Fig. 6C, Sup. Fig. 6C). Next studies were conducted to ascertain if increase in ERK phosphorylation was driving fisetin-induced apoptosis in melanoma cells. We found that ERK inhibition did not abrogate caspase-3 cleavage in fisetin treated cells (Fig. 6D, Sup. Fig. 6D). We further compared the effects of fisetin and vemurafenib in athymic nude mice implanted with 451Lu melanoma cells. The animals were divided into three cohorts, each with 6 animals that were administered fisetin/DMSO (intraperitoneally) or vemurafenib (orally). A smaller average tumor volume was observed in mice treated with fisetin and vemurafenib. Upon termination of the study, tumor volumes evaluated between the three groups showed no statistical difference in tumor inhibition between animals receiving fisetin or vemurafenib (p = 0.2). Notably, a significant decrease in tumor volumes was observed in animals receiving fisetin or vemurafenib when compared to DMSO treated controls (Fig. 6E). In the control group, the average tumor volume of 1179.7 mm^3^ was reached at day 35, while mice receiving fisetin (1 mg/animal) and vemurafenib (2 mg/animal) had an average tumor volume of 789.3 (p = 0.05) and 641.7 mm^3^ (p = 0.002) respectively. Similar to our *in vitro* studies in A375 melanoma cells, 451Lu xenograft lysates treated with fisetin and vemurafenib showed dephosphorylated RSK and decreased YB-1 levels (Fig. 6F, Sup. Fig. 6E). However, in contrast to our *in vitro* data, we did not observe increased ERK phosphorylation in fisetin-treated mice. Animals treated with fisetin showed reduced phosphorylation of ERK1/2 as was observed in 3-D melanoma cultures exposed to fisetin for 16 days (Fig. 6F, Sup. Fig. 6F).

## Discussion

**Figure 7:**
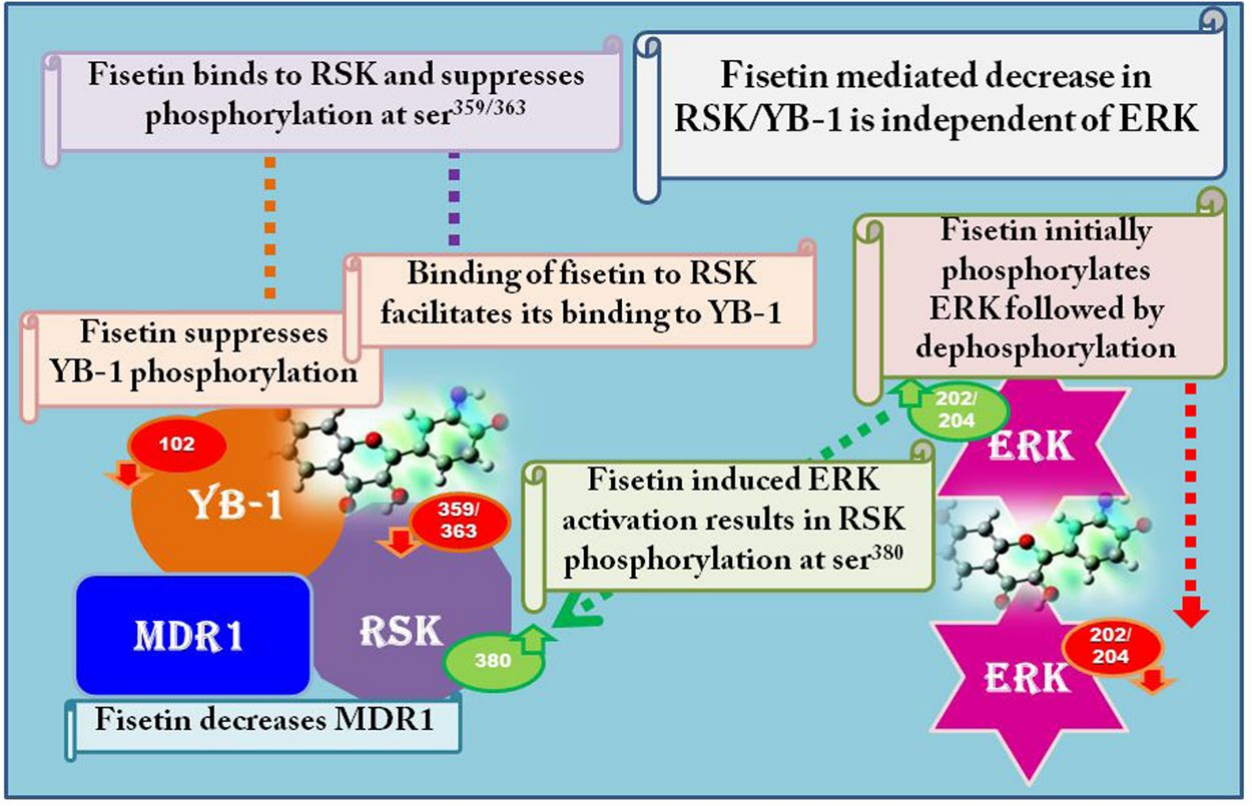
Schematic diagram depicting the proposed mechanism of action of fisetin: SPR studies showed that interaction of fisetin with RSK2 promotes its binding to YB-1. Fisetin mediated downregulation of YB-1/RSK signaling is associated with a decrease in MDR1. Fisetin-treated cells showed an initial increase in ERK1/2 phosphorylation at threonine^202^/tyrosine^204^ followed by a sustained decrease, suggestive of a biphasic response. Fisetin mediated decrease in RSK/YB-1 is independent of ERK. Inhibition of ERK did not interfere with RSK phosphorylation at threonine^359^/serine^363^ residues in fisetin-treated cells. However, fisetin mediated increase in RSK serine^380^ phosphorylation was abrogated with ERK inhibition suggesting that ERK was driving phosphorylation at this residue.

New treatment options are constantly being explored to improve the prognosis of malignant melanoma, an aggressive and notoriously chemoresistant cancer^29^. The specificity paradigm that ascribed central relevance to achieving target specificity of drug candidates is now being reconsidered. Indeed there is considerable evidence that polypharmacological drug behavior is often responsible for improved therapeutic efficacy. Studies show that active compounds may interact with multiple targets, which in most cases, are related to each other^30^. Our lab and others have investigated and established the efficacy of dietary flavonoid fisetin in inhibiting melanoma growth in various experimental models, both *in vitro* and *in vivo*^13^. Defining the precise mechanism of action of fisetin remains a challenge due to multiple targets that may reside within sequentially regulated phosphorylation cascades with several interacting partners. Ongoing studies have delineated various signaling pathways involved in growth and proliferation that are evident targets of fisetin^13^. We demonstrated previously that fisetin inhibited YB-1 and markers of EMT in prostate cancer cells. Here we validated the effect of fisetin on YB-1 and evaluated its interaction with RSK in BRAF-mutant melanoma cells. The insights gained from this study include (i) fisetin mediated inhibition of YB-1 extends to other cancer cell types including melanoma and ovarian cancer cells (ii) fisetin binds to RSK2 avidly and this complex augments the binding of fisetin to YB-1 (iii) fisetin suppresses MDR1 downstream of YB-1 but inhibition does not translate into decreased substrate efflux (iv) fisetin mediated RSK suppression is independent of its effect on ERK signaling (Fig. 7).

Disruption of YB-1 phosphorylation by site-directed mutagenesis suppressed tumor growth in various tumor models^31^. Cell permeable interference peptides acting as molecular decoys and kinase inhibitors are being explored to block activation of endogenous YB-1^32^. While a considerable proportion of its oncogenic effect is thought to be facilitated by its transcriptional activity mediated by serine^102^ phosphorylation, YB- 1 can suppress or stimulate translation. It was noted that enhanced cytoplasmic expression of YB-1 in breast cancer cells may result in induction of tumor dormancy which can be reverted upon re-phosphorylation of YB-1^33^. Thus targeting total YB-1 levels through gene silencing studies was suggested to be a better approach for decreasing YB-1 levels and inhibiting tumor growth and invasion^34^. Our studies in monolayer and 3-D melanoma cell cultures showed that fisetin treatment to melanoma cells achieves the desired decrease in YB-1 abundance by targeting YB-1 at both protein and mRNA levels (Fig. 1).

There exists significant cross talk between YB-1 and RSK, crucial signaling molecules implicated in tumor growth, EMT and invasion^35–37^. Small-molecules that modulate interactions between specific proteins represent a promising avenue for therapeutic intervention in a variety of settings. Binding of the compound to the proteins may result in change in conformation that is distinct from the unbound or protein-bound conformations^38^. In accordance with cell-free data that provided evidence that fisetin binds avidly to RSK and inhibits its kinase activity, we observed a decrease in RSK phosphorylation at threonine^359^/serine^363^ residues in cells treated with fisetin. In fact, both fisetin and the RSK inhibitor BI-D1870 exerted analogous effects on RSK phosphorylation^25^. Future studies will describe whether fisetin and BI-D1870, an ATP-competitive inhibitor, share common binding sites or target different areas on the kinase. Employing SPR studies, we evaluated the binding of fisetin with different isoforms of RSK and found strong binding with RSK2. We observed that addition of fisetin in the presence of RSK2 facilitated its binding to YB-1. We postulate formation of a ‘ternary complex’ between RSK2/YB-1/fisetin, where fisetin stabilizes the interaction between RSK2 and YB-1 by acting at the interface between the two kinases. The interaction between RSK and YB-1 reportedly culminates in phosphorylation and activation of YB-1^24,39^. In our model, however, binding of RSK2 with YB-1 does not translate into increased activity of YB-1. NMR studies elucidating folding dynamics of proteins indicate that binding of a ligand at a specific site may bias the conformational equilibrium toward a structure in which the active site may or may not be active^40^. Thus, binding of the allosteric effector by altering the conformation of the protein may well result into an altered functional output^40^. It is tempting to speculate that fisetin in a complex with YB-1 and RSK induced allosteric modifications in YB-1 such that RSK is unable to phosphorylate it at the serine^102^ residue. This hypothesis is consistent with our previous observations where we showed that fisetin not only binds within the CSD of YB-1 but also alters its topology^21^. At this stage we have not explored concomitant interactions, yet we cannot exclude that this complex may also interfere with phosphorylation of RSK at threonine^359^/serine^363^ by upstream kinases. The fact that ERK mediated phosphorylation of RSK at serine^380^ residue remained unaffected by this complex indicates that this effect may be specific to the afore-mentioned site. It is interesting to note that fisetin did not affect RSK protein levels as opposed to YB-1 where a reduction in phosphorylation was accompanied by suppressed protein and mRNA levels. This suggests that fisetin may regulate YB-1 not only through modulation of phosphorylation through allosteric modifications but may also interact with YB-1 at the mRNA level to effect a conformational shift. Additional studies will further clarify mechanism(s) involved in fisetin mediated suppression of YB-1/RKS axis.

The well-characterized multidrug transporter MDR1 is a major component of the energy-dependent efflux system, responsible for removal of chemotherapeutics out of tumor cells^41^. Although indisputable that MDR1 effluxes xenobiotics from cells, insights into the biological function of MDR1 and related transporter molecules suggest additional roles in chloride channel activity, cholesterol metabolism and immune cell function. Its involvement in regulation of cell differentiation, proliferation, and survival has been documented^42^. MDR1 was found to protect cells from apoptosis induced by a wide array of cell death stimuli that relied on activation of intracellular caspases for full function^42,43^. Recently, it was shown that MDR1 associated with the calcium-dependent phospholipid-binding protein Annexin A2 to promote invasion in multidrug-resistant breast cancer cells^44,45^. In our studies we observed that fisetin downregulated MDR1 both at transcription and translational levels however the decrease in MDR1 was not associated with intracellular accumulation of MDR1 substrates. It is probable that MDR1 partakes in YB-1/RSK driven EMT and cell survival signals. Fisetin mediated suppression of melanoma cell proliferation and invasion may be linked to its inhibitory effect on YB-1/RSK signaling that in turn regulates MDR1 thereby inhibiting tumor growth.

An important aspect of our work was to elucidate the regulation of ERK signaling by fisetin in melanoma cells. Studies in 3-D and animal models representative of long term treatment response showed dephosphorylation of ERK with fisetin treatment. This correlated with data acquired from cell-free studies where fisetin was found to be a potent inhibitor of ERK activity. Interestingly, time course studies in monolayer melanoma cells indicate a biphasic response where suppressed ERK activity was preceded by an initial increase in ERK phosphorylation. We have previously demonstrated that fisetin treatment results in increased nitric oxide levels in melanoma cells^18^. In certain cell types, nitric oxide has been shown to promote ERK signaling via redox reactions^46^. It can be surmised that the initial increase in ERK observed in fisetin treated cells may be associated with enhanced nitric oxide levels, however additional studies are warranted. Importantly, our *in vitro* data unambiguously shows that suppression of RSK phosphorylation by fisetin is not mediated through negative regulation of ERK signaling. Moreover, fisetin-induced cell death is independent of ERK signaling as shown in Fig. 6. Comparative studies of fisetin with vemurafenib underline the efficacy of fisetin and shed light on the molecular mechanisms involved in inhibition of melanoma growth. Going forward a combinatorial study employing low doses of vemurafenib and fisetin can provide powerful evidence to examine the dietary flavonoid in pre-clinical trials and subsequently in clinical settings.

## Electronic supplementary material

Supplementary Information
