# Supplementary material for: Fisetin targets YB-1/RSK axis independent of its effect on ERK signaling: insights from in vitro and in vivo melanoma models
Source: Sci Rep. 2018 Oct 24;8:15726. doi: 10.1038/s41598-018-33879-w (PMC6200766; doi:10.1038/s41598-018-33879-w)
Supplement: Supplementary file 1 — Supplementary Information [file 41598_2018_33879_MOESM1_ESM.pdf]

Fisetin targets YB-1/RSK axis independent of its effect on ERK signaling: insights from *in vitro* and *in vivo* melanoma models

Mario Sechi[3], Rahul K. Lall[1], Saheed O Afolabi[1], Anant Singh[1], Dinesh C. Joshi[2], Shing-Yan Chiu[2], Hasan Mukhtar and Deeba N. Syed[1]

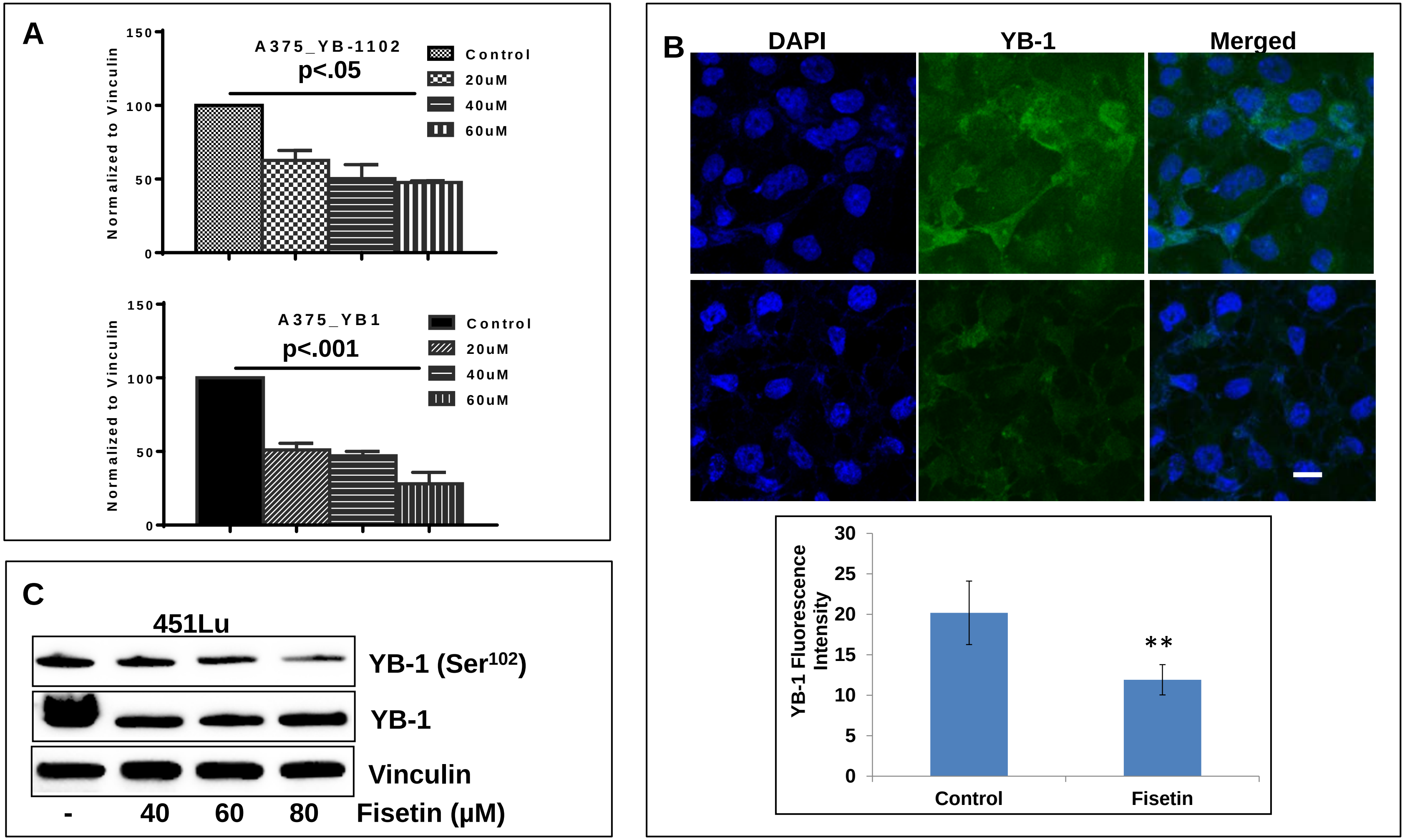

SupFig.1: **Fisetin inhibits YB-1 in BRAF mutant melanoma cells** (A) Densitomeric analysis of phosphorylated and total YB-1 expression in fisetin treated cells. Whole cell lysates of A375 melanoma cells, treated with fisetin (20-60µM:24h) were analyzed for p-YB-1and YB-1 protein expression. Relative density of the bands was normalized to vinculin. (B) (*Top*) Representative micrographs (20x) showing immunofluorescence for YB-1 (green) in A375 cells with or without fisetin (60µM:24h). Images were captured by a confocal microscope. Scale bar, 20µm. (*Bottom*) Histogram represents relative fluorescent intensity of YB-1, scored using ImageJ in A375 cells treated with/without fisetin. (C) Whole cell lysates of 451Lu melanoma cells treated with fisetin (40-80µM:24h) were analyzed for p-YB-1 and YB-1 expression. Equal loading was confirmed by reprobing for vinculin.

Syed et al

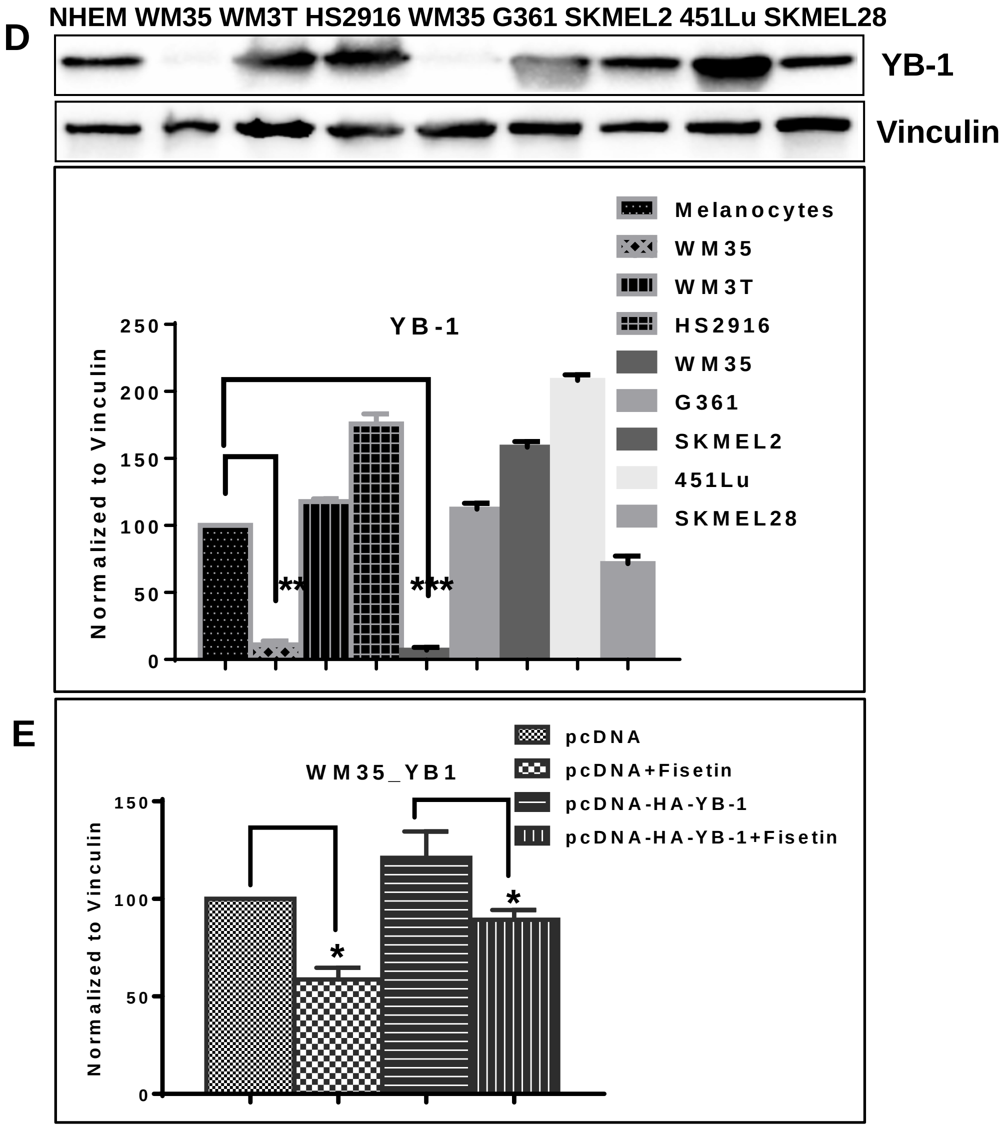

SupFig.1: **Fisetin inhibits YB-1 in BRAF mutant melanoma cells:** (D) Whole cell lysates of melanoma cell lines were analyzed for basal YB-1 expression. WM35 melanoma cells from two different passages were loaded twice. Equal loading was confirmed by reprobing for vinculin. (*top*). Relative density of the bands normalized to vinculin (*bottom*) (E) Densitometric analysis of YB-1 expression in WM35 melanoma cells. WM35 melanoma cells transfected with pcDNA-HA-YB-1 and treated with fisetin (60µM:24h) were analyzed for YB-1 expression. Relative density of the bands were normalized to vinculin. Data shown are representative of three independent experiments with similar results.

Syed et al

F

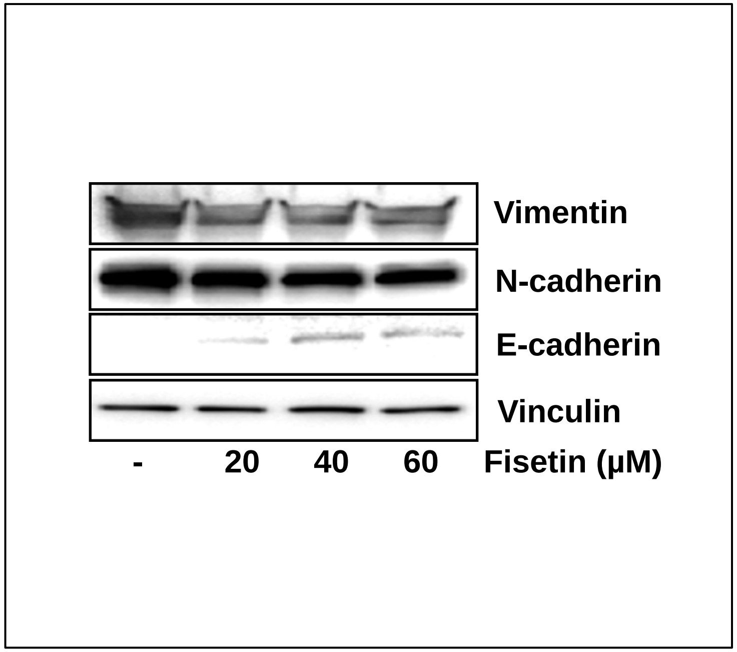

G

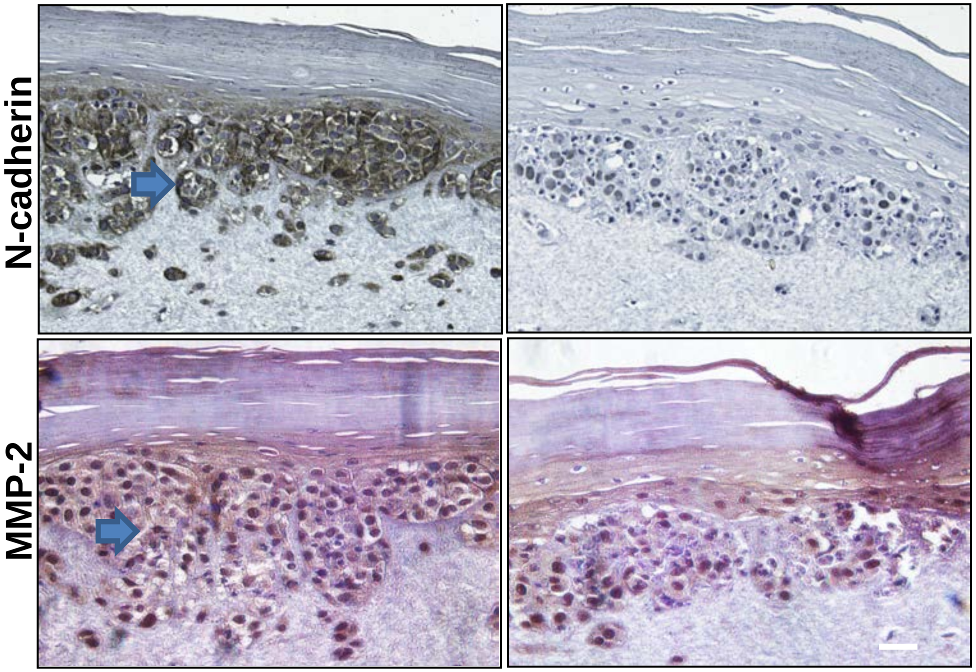

SupFig.1: **Fisetin inhibits YB-1 in BRAF mutant melanoma cells:** (F) Whole cell lysates of A375 melanoma cells treated with fisetin (20-60µM:24h) were analyzed for EMT regulatory proteins. Equal loading was confirmed by reprobing for vinculin. (G) Representative micrographs (10x) showing MMP2 and N-cadherin expression in A375 melanoma constructs treated with fisetin (80µM), harvested at day 12 post treatment. Scale bar, 20µm. Data shown are representative of three independent experiments with similar results.

Syed et al

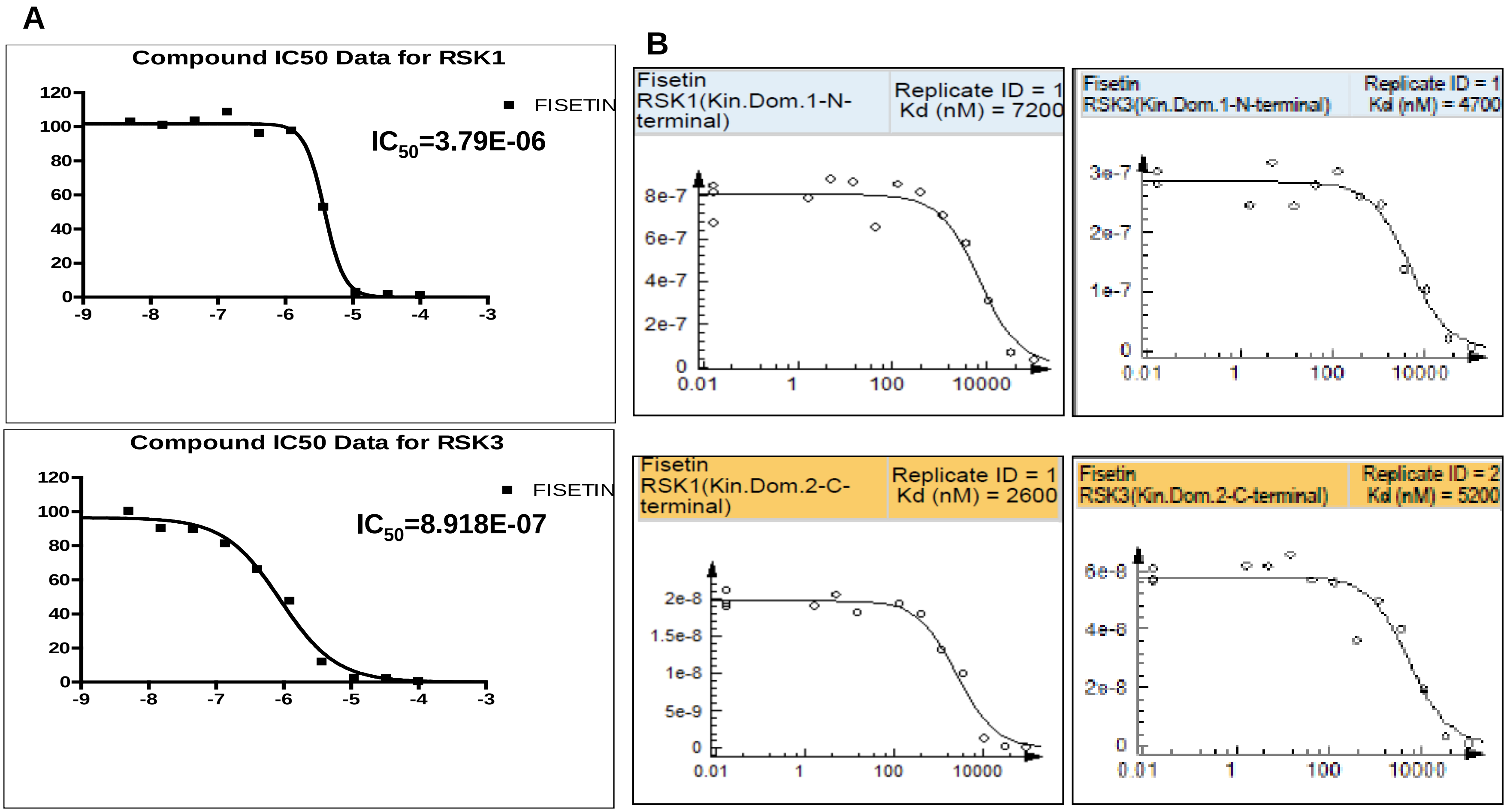

SupFig.2: **Fisetin binds to RSK and suppresses its kinase activity (A)** Representative curves of % kinase activity for RSK isoforms. Fisetin was tested for kinase activity inhibition against RSK1 and RSK3 in 10-dose IC_{50} mode with 3-fold serial dilution starting at 100μM. Reactions were carried out at 30μM ATP. **(B)** Representative curves of competition binding assay for RSK1 and RSK3. An 11-point 3-fold serial dilution of fisetin was prepared in 100% DMSO at 100X final test concentration and diluted to 1X in the assay. Kds were determined using a top concentration of 100μM. The amount of kinase measured by qPCR (signal; *y*-axis) is plotted against the corresponding compound concentration in log10 scale (*x*-axis).

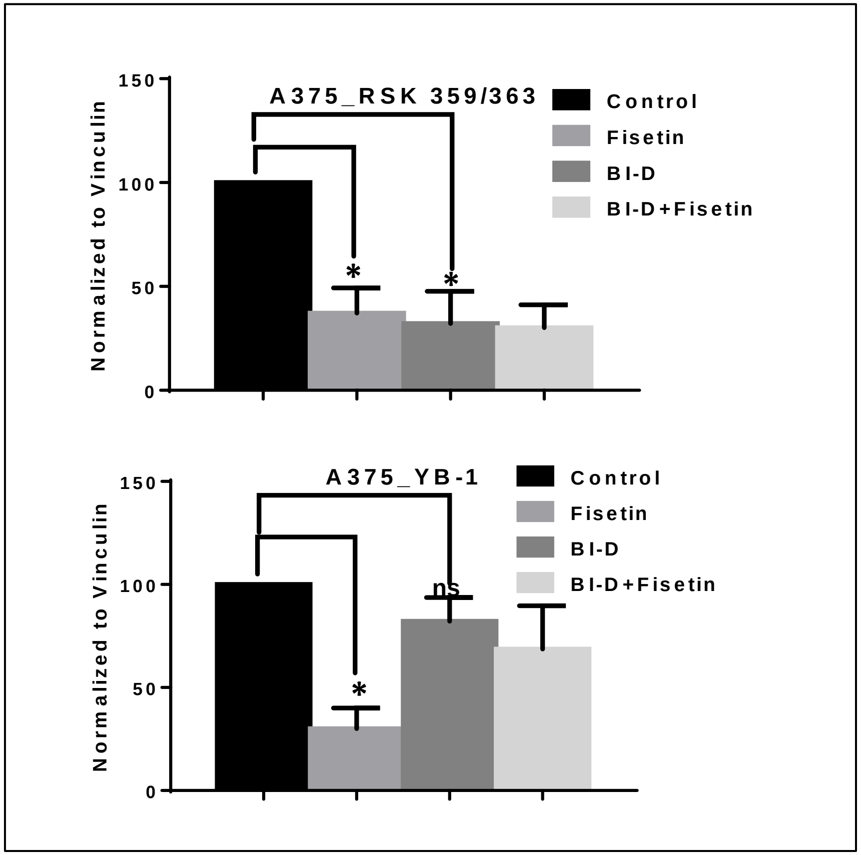

SupFig.3: **Fisetin/RSK2 complex augments its binding to YB-1:** Whole cell lysates of fisetin-treated A375 melanoma cells (60µM:24h) with/without RSK inhibitor BI-D1870 were analyzed for RSK (top) and YB-1 expression (*bottom*). Relative density of the bands were normalized to vinculin. Data shown are representative of three independent experiments with similar results.

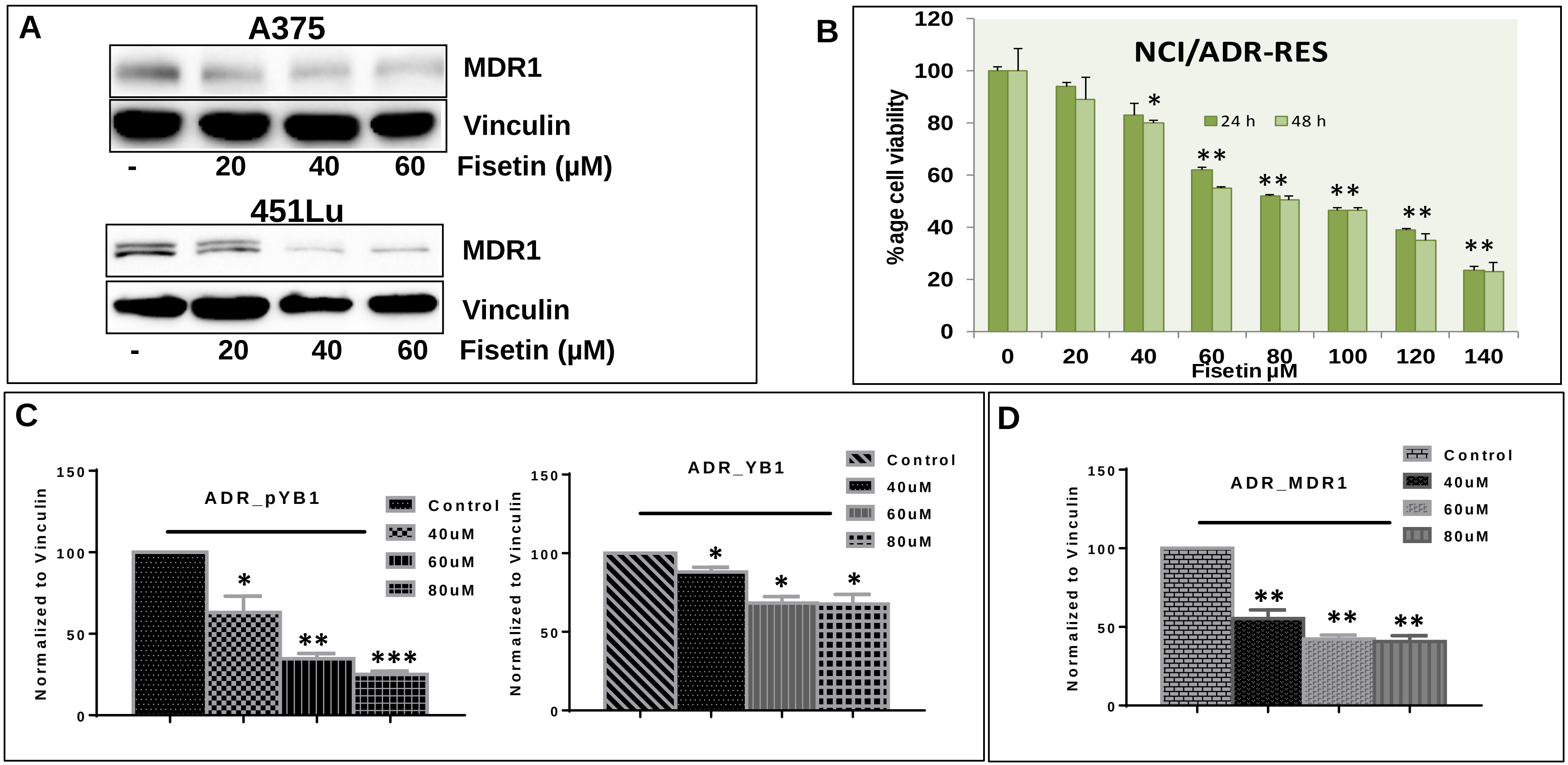

SupFig.4: **Fisetin induced downregulation of YB-1/RSK signaling is associated with decrease in MDR1 (A)** Whole cell lysates of fisetin treated A375 and 451Lu melanoma cells (24 h) were analyzed for MDR1 expression. Equal loading was confirmed by reprobing for vinculin. **(B)** Cell viability studies in fisetin-treated NCI-ADR-Res ovarian cancer cells, 24 and 48h post treatment was assessed by MTT assay. The data expressed as the percentage cell viability represent the mean±SE of three experiments. **(C&D)** Whole cell lysates of NCI/ADR-Res ovarian cancer cells treated with fisetin (40-80µM:24h) were analyzed for p-YB-1, YB-1 and MDR1 expression. Histograms represent relative density of bands normalized to vinculin. Data shown are representative of three independent experiments with similar results.

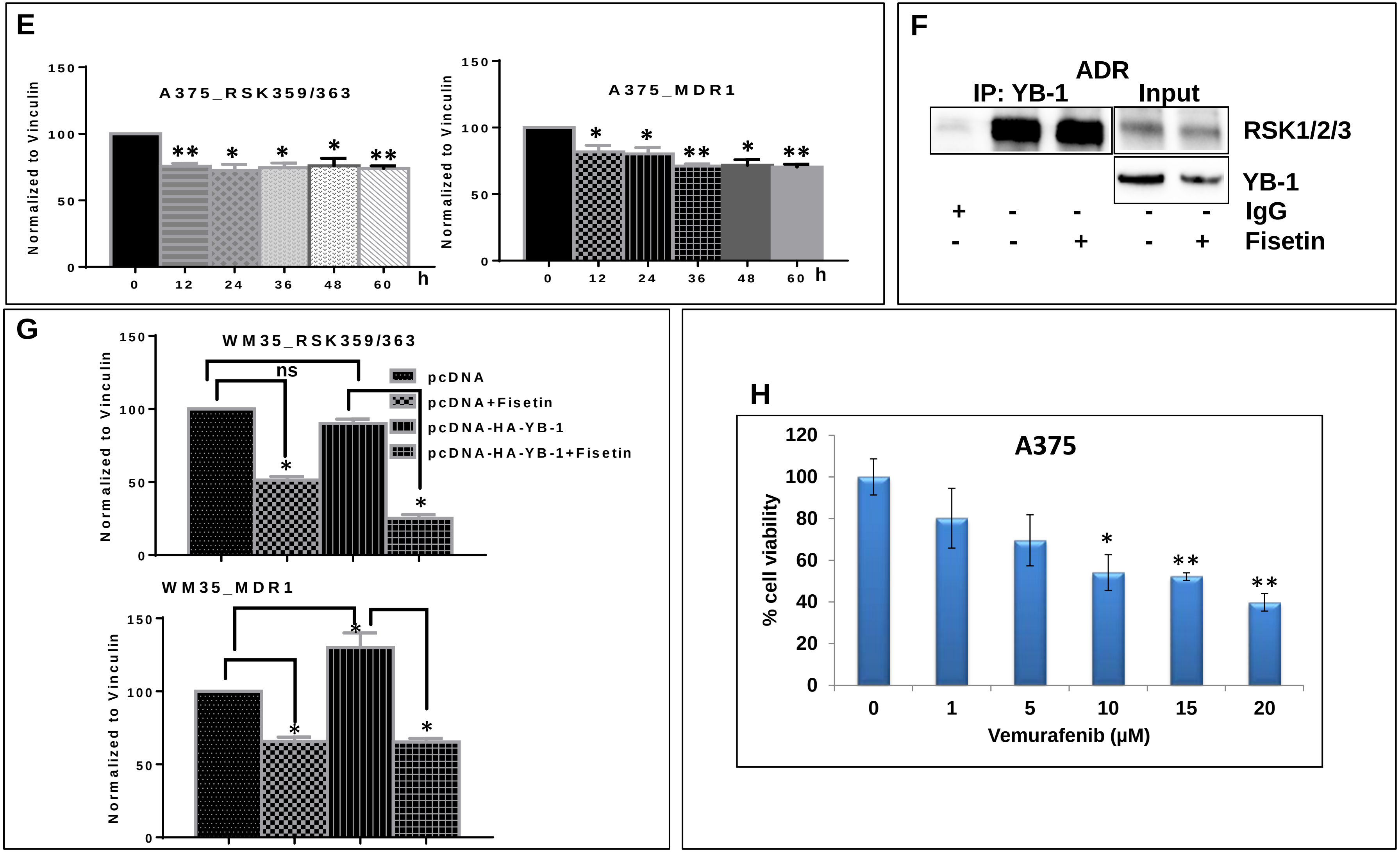

SupFig.4: **Fisetin induced downregulation of YB-1/RSK signaling is associated with decrease in MDR1 (E)** Whole cell lysates of A375 melanoma cells treated with fisetin (20-60µM:24h) were analyzed for p-RSK and MDR1 expression at specified time points. Histograms represent relative density of bands normalized to vinculin. **(F)** Equal amounts of NCI-ADR cell lysates treated with/without fisetin (60µM:24h) were immunoprecipitated with YB-1 antibody followed by western blot analysis for RSK1/2/3 and YB-1. **(G)** WM35 melanoma cells transfected with pcDNA-HA-YB-1, treated with fisetin (60µM:24h) were analyzed for p-RSK and MDR1 expression. Histograms represent relative density of bands normalized to vinculin. Data shown are representative of three independent experiments with similar results. (**H**) A375 melanoma cells were treated with varying doses of vemurafenib and MTT assay was performed after 24h. The data expressed as the percentage of cell viability represent the mean±SE of three experiments.

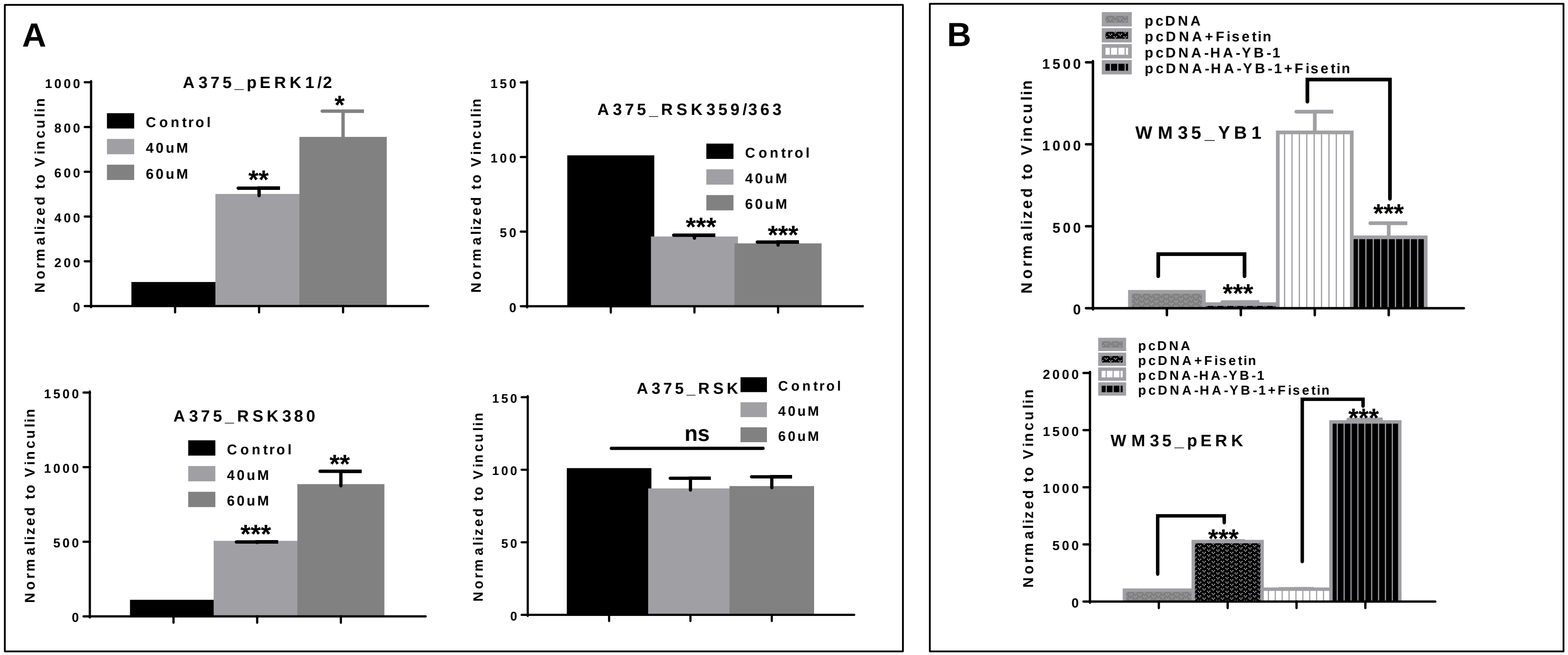

SupFig.5: **Fisetin mediated decrease in RSK/YB-1 does not require suppression of ERK signaling (A)** Whole cell lysates of A375 cells treated with fisetin (40-60µM:24h) were analyzed for ERK1/2 and RSK protein expressions. Histograms represent relative density of the bands normalized to vinculin. **(B)** WM35 melanoma cells, transfected with pcDNA-HA-YB-1, treated with fisetin (60µM:24h) were analyzed for p-ERK1/2 and YB-1 expression. Histograms represent relative density of the bands normalized to vinculin. Data shown are representative of three independent experiments with similar results.

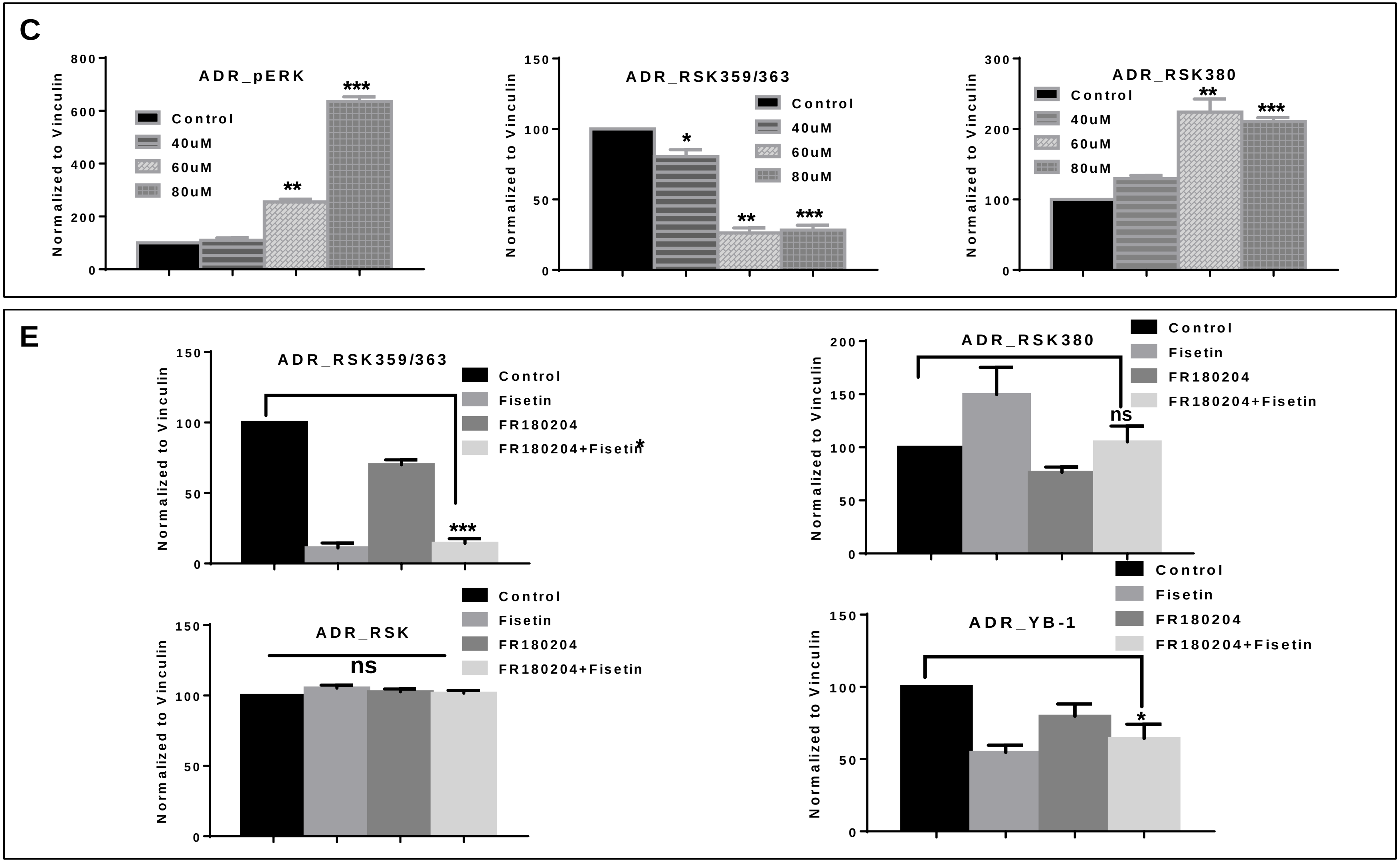

SupFig.5 **Fisetin mediated decrease in RSK/YB-1 does not require suppression of ERK signaling (C)** Whole cell lysates of NCI/ADR-RES ovarian cancer cells treated with fisetin (40-80µM:24h) were analyzed for phosphorylated RSK and ERK1/2 protein expressions. Histograms represent relative density of the bands normalized to vinculin. **(E)** Whole cell lysates of NCI/ADR-RES ovarian cancer cells, treated with fisetin (60µM:24h) and/or ERK1/2 inhibitor FR180204 (10µM) were analyzed for phosphorylated RSK and YB-1 protein expressions. Histograms represent relative density of the bands normalized to vinculin. Data shown are representative of three independent experiments with similar results.

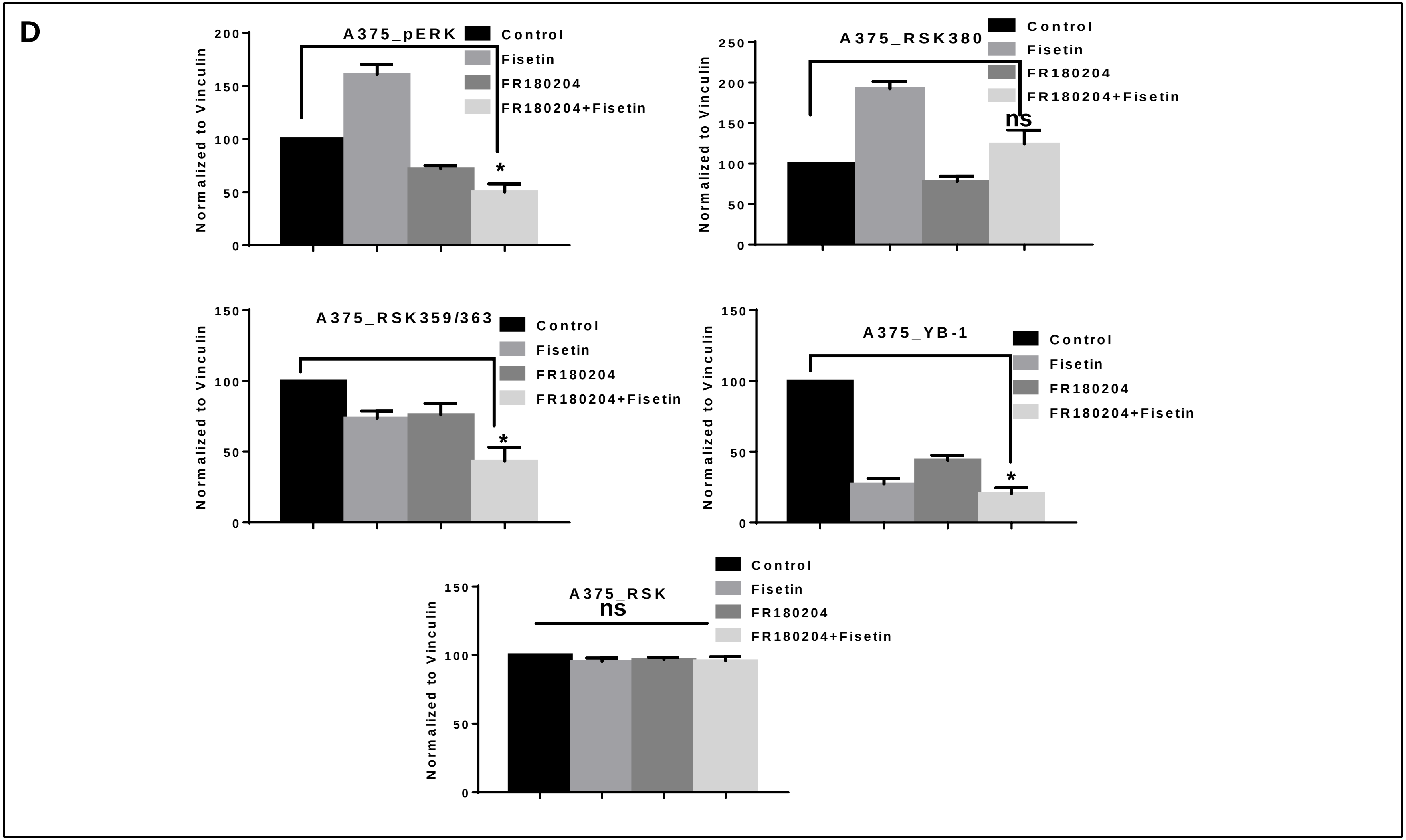

SupFig.5 **Fisetin mediated decrease in RSK/YB-1 does not require suppression of ERK signaling (D)** Whole cell lysates of A375 melanoma treated with fisetin (60µM:24h) and/or ERK1/2 inhibitor FR180204 (10µM) were analyzed for phosphorylated RSK and YB-1 protein expressions. Histograms represent relative density of the bands normalized to vinculin. Data shown are representative of three independent experiments with similar results.

A

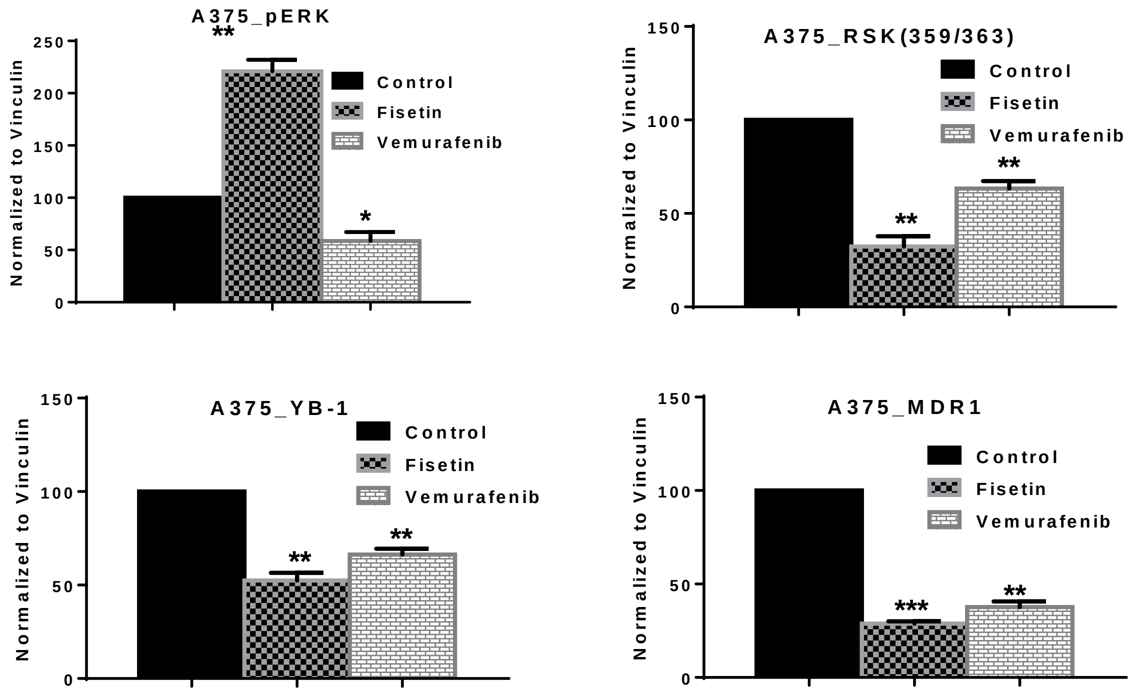

SupFig.6 **Comparable inhibition of melanoma tumor growth by fisetin and vemurafenib albeit through divergent regulation of ERK signaling (A)** Whole cell lysates of A375 melanoma cells treated with fisetin (60μM) or vemurafenib (10μM) for 24h were analyzed for phosphorylated RSK, ERK1/2, total YB-1 and MDR1. Histograms represent relative density of the bands normalized to vinculin. Data shown are representative of three independent experiments with similar results.

B

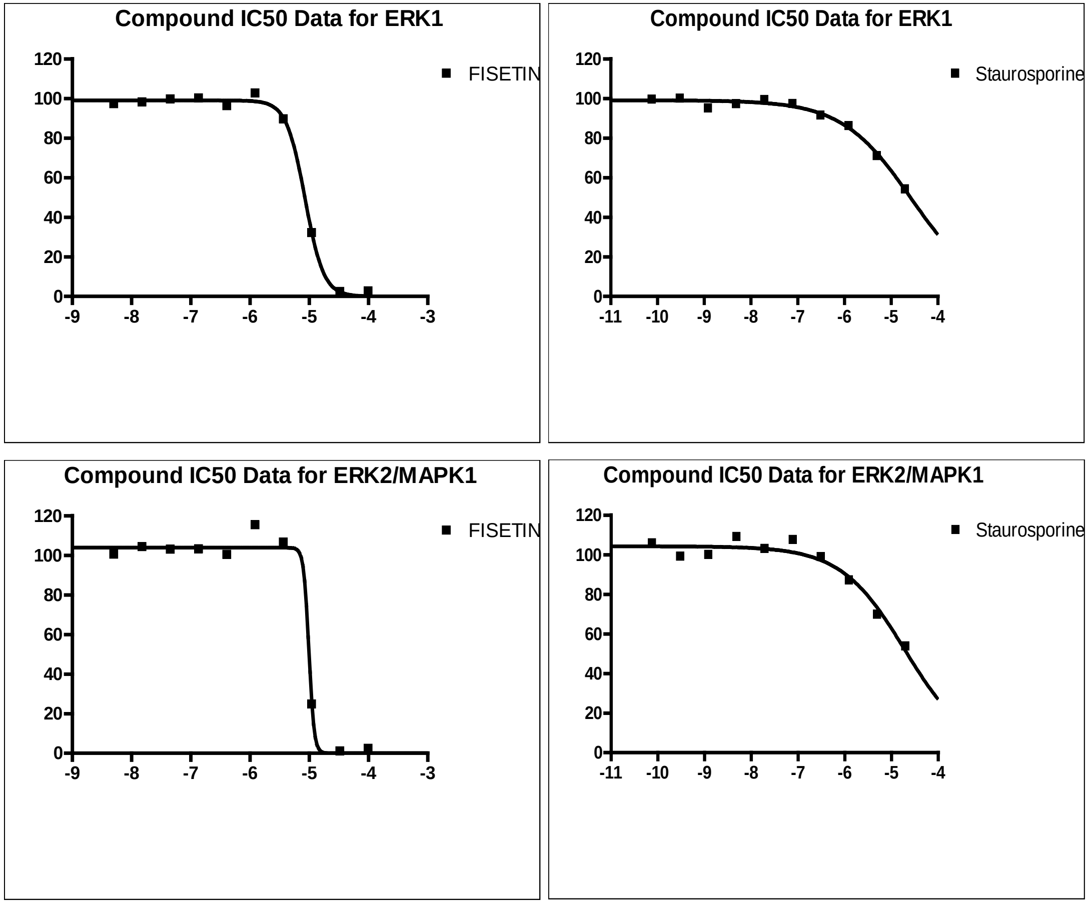

SupFig.6 **Comparable inhibition of melanoma tumor growth by fisetin and vemurafenib albeit through divergent regulation of ERK signaling (B)** Representative curve of % kinase inhibitory activity for ERK1/2 with fisetin and staurosporine. Fisetin was tested for kinase activity along with reference compound staurosporine in 10-dose IC_{50} mode with 3-fold serial dilution starting at 100μM. Staurosporine was tested in 10-dose IC_{50} mode with 4-fold serial dilution starting at 20μM. Reactions were carried out at 30μM ATP.

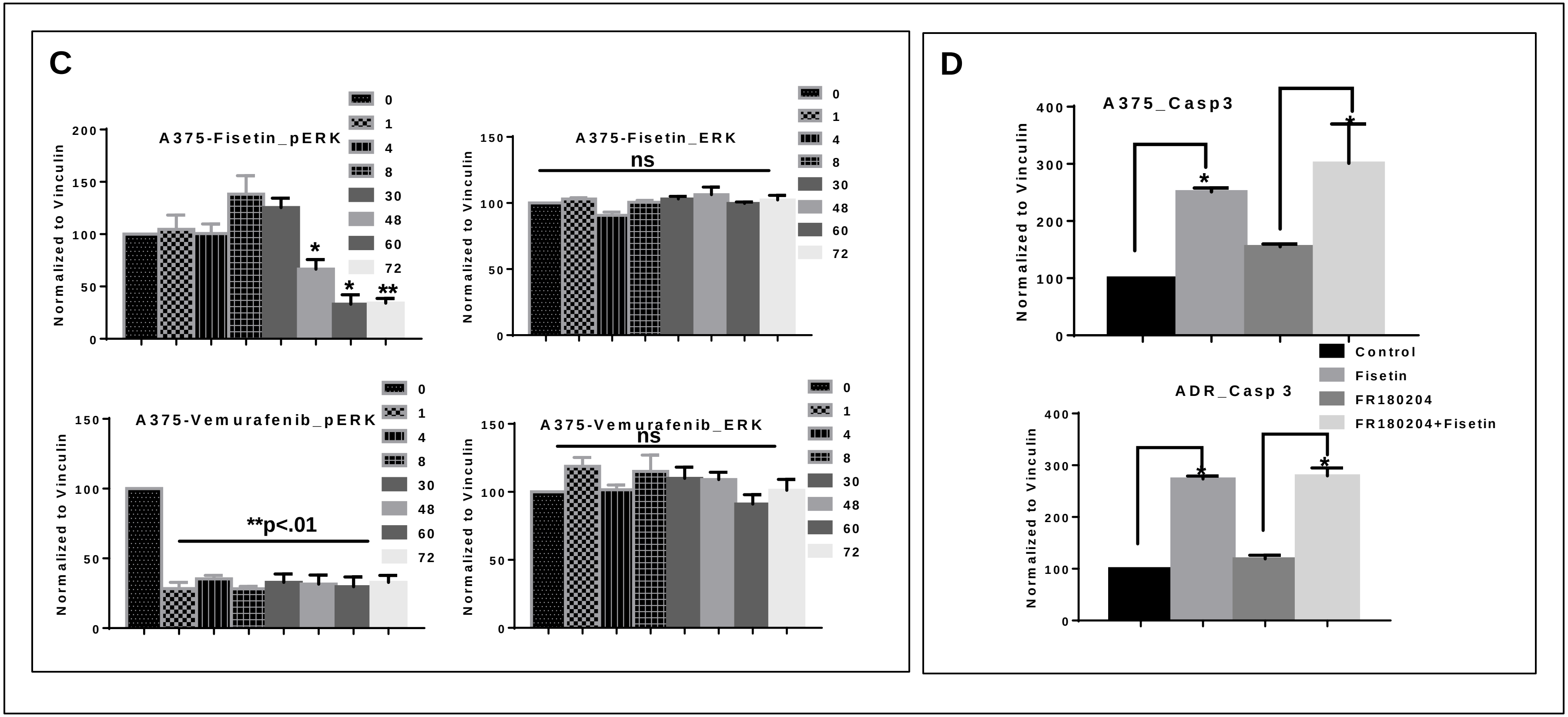

SupFig.6 **Comparable inhibition of melanoma tumor growth by fisetin and vemurafenib albeit through divergent regulation of ERK signaling (C)** Whole cell lysates of A375 melanoma cells treated with fisetin (60µM) or vemurafenib (10µM) for specified time points were analyzed for phosphorylated and total ERK1/2. Histograms represent relative density of the bands normalized to vinculin. **(D)** Whole cell lysates of A375 melanoma and NCI/ADR-RES ovarian cancer cells, treated with fisetin (60µM) and/or ERK1/2 inhibitor FR180204 (10µM) for 24h were analyzed for cleaved caspase-3 expression. Histograms represent relative density of the bands normalized to vinculin. Data shown are representative of three independent experiments with similar results.

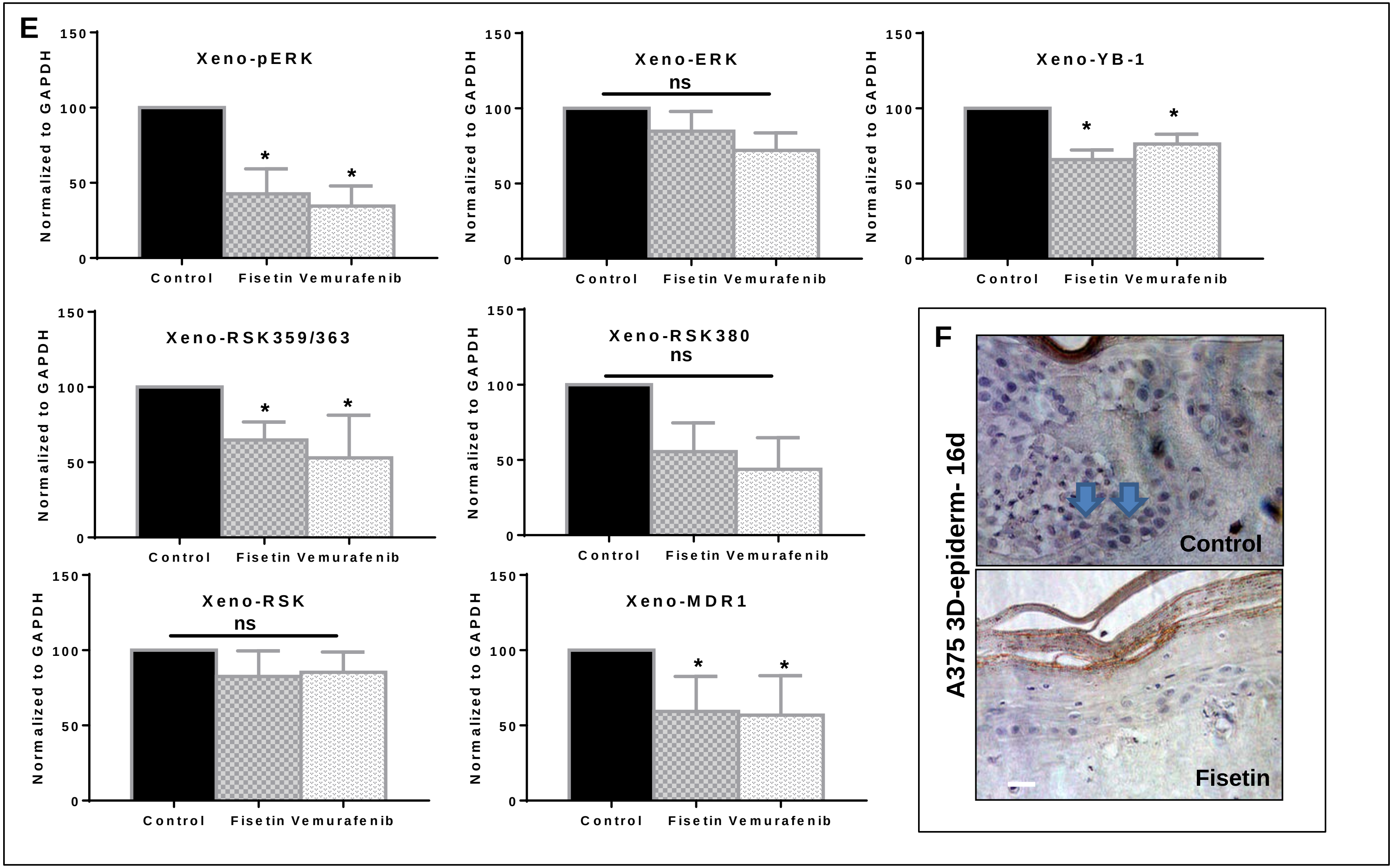

SupFig.6 **Comparable inhibition of melanoma tumor growth by fisetin and vemurafenib albeit through divergent regulation of ERK signaling (E)** Whole cell lysates of tumor tissues from DMSO/fisetin/vemurafenib-treated mice analyzed by western blot. Histograms represent relative density of the bands normalized to GAPDH. **(F)** Representative micrographs (10x) showing pERK1/2 expression (arrow) in A375 melanoma constructs treated with/without fisetin (80µM), harvested at day 16 post treatment. Scale bar, 10 µm. Data represent samples from each group repeated twice with similar results.
